# Wearable Cooling Textiles of Thermal Conduction and Sweat Transfer for Personal Thermal Management

**DOI:** 10.1002/advs.202523061

**Published:** 2026-01-26

**Authors:** Jiajing Zhang, Jiahao Xu, Chunhua Zhang, Liangjun Xia, Xin Liu, Weilin Xu

**Affiliations:** ^1^ State Key Laboratory of New Textile Materials and Advanced Processing Wuhan Textile University Wuhan P. R. China; ^2^ College of Textile Science and Engineering Zhejiang Sci‐Tech University Hangzhou P. R. China

**Keywords:** double‐layer structure, personal thermal comfort textiles, thermal conductivity, unidirectional moisture conductivity

## Abstract

Given rising global temperatures, advanced protective textiles hold significant potential to enhance productivity, conserve energy, and improve personal thermal comfort. Extensive research has shown that both thermal conduction and moisture management are equally critical in determining the comfort performance of textiles. Here, we propose a wearable cooling textile (WCT) that integrates thermal conduction and unidirectional moisture transfer through a 3D laminating method. Using thermally conductive fibers. By constructing a 3D thermal conductive network and a double‐layer *Janus* wetting structure, boron nitride nanosheet (BNNS) attached to the 3D framework, endowing the textile with omnidirectional heat dissipation (thermal conductivity of 0.315 W·m^−1^·K^−1^), unidirectional moisture‐wicking properties (transport index of 476%), and a high water evaporation rate (WER, 5209.92 g/m^2^/day). Compared with commercial cotton fabrics, the WCT reduces temperature by up to 4.1°C due to its cooling mechanism. Thus, this work provides a promising strategy for developing textiles that can effectively regulate both heat and moisture under diverse and demanding environmental conditions.

## Graphical abstract

This study constructs a three‐dimensional thermal‐conductive pathway using biomass‐based fibers. The thermally conductive composite fibers were designed into *Janus*‐structured fabrics. The wearable cooling textile exhibits unidirectional moisture‐wicking properties (476% index), a high water evaporation rate (5209.92 g/m^2^/day), and effective skin‐cooling performance (reducing temperature by 4.1°C compared to traditional textiles), meeting key demands for personal thermal management.

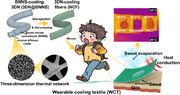

## 1 Introduction

The greenhouse effect's intensifying summer heatwave causes physiological and psychological problems threats to human health and have a detrimental influence on the socioeconomic systems and labor productivity [1, 2]. Functional designed textiles that regulate the thermal and hygroscopic microclimate around human skin show significant potential to enhance thermal comfort. Such textiles offer an effective means to a promote human well‐being, conserve energy, and generate economic benefits [3, 4]. Recently, thermal comfort textiles have attracted considerable interest for they ability to balance skin temperature and sweat evaporation, thereby effectively regulating body comfort [5, 6]. Unlike common textiles, thermal comfort textiles are worn close to the skin and can modulate the microclimate of the human skin, offering personal comfort without relying on large‐scale external heating or cooling systems. Researchers have developed textiles with intriguing self‐cooling or heat‐retaining properties by constructing special thermally conductive composite textiles or by designing structures that control thermal radiation. However, thermal management alone is often insufficient [7, 8, 9].

If perspiration is not effectively removed, wearers continue to feel hot and sticky. Therefore, another way to maintaining cool, dry skin is to use moisture‐management fabrics with altered wettability or fiber architectures that enhance sweat wicking and evaporation [10]. However, uniformly treated fabrics, such as those with profiled fibers or homogeneous hydrophilicity, cannot transport moisture directionally, often leaving sweat trapped on the skin side. Advances in *Janus* membrane technology have enabled directional liquid transport [11, 12, 13]. A *Janus* membrane's asymmetric properties, such as wettability or structural gradients, create driving forces that propel liquid movement irreversibly across the membrane. Based on this principle, directional moisture management textiles have been developed by making the inner (skin) side hydrophobic and the outside side hydrophilic, or by enhancing capillary force from the inner to the outer side [14, 15]. Such *Janus* designs allow sweat to be rapidly transported away from the skin and spread outwardly for faster evaporation, creating a cooler and drier micro‐environment. This targeted cooling and drying effect is a practical way to preserve individual comfort. Nevertheless, real life scenarios are more complex, for instance, moving frequently between hot and cool environment is common [16, 17]. Therefore, developing smart textiles that synergistically dissipate both heat and moisture is essential for achieving optimal comfort.

Clothing acts as a protective barrier and plays a vital role in regulating body temperature and moisture. Integrating cooling functionality into textiles presents a promising strategy for practical applications. Personal cooling textiles can provide localized cooling to specific body areas and be adapted to meet diverse environmental requirements for thermal comfort [18]. Generally, personal cooling textiles are categorized as either active or passive systems. Passive cooling textiles can be further divided into radiative cooling, thermal conductive cooling, and evaporation cooling textiles [19, 20, 21]. Among these, thermally conductive cooling and radiative cooling textiles rely on environmental conditions, while evaporation cooling textiles enhance cooling by accelerating sweat evaporation from the skin. *Janus* structures have been applied to design thermally adaptive textiles through specialized weaving techniques. Bai et al. designed an interlocking double‐layer textile (Foam‐TEX) via back‐weft weaving to construct a gradient vapor transmission channel, achieving a low thermal conductivity of 0.039 W·m^−1^·K^−1^ and effectively preventing heat loss in cold, sweaty conditions [22]. Wang et al. developed a double‐sided synergetic *Janus* textile capable of reversible unidirectional water transport and adjustable thermal convection, showing 50% faster water evaporation and a 1.2°C–2.3°C lower temperature than cotton [23]. Zhao et al. designed a smart treble‐weaving electronic textile (TWET) with superior personal radiative cooling performance, reducing temperature by about 10°C compared to conventional cotton [24]. Enhancing heat dissipation performance remains critical, yet the internal mechanisms so involved are multifaceted. Integrating multiple cooling mechanism while improving the efficiency of each mode to achieve effective all‐scenario human body cooling remains a considerable challenge. Several researchers have proposed that innovative textile‐weaving methods [25] or fabric assembly strategies [26, 27] can simultaneously address thermal conduction and moisture management to meet personal thermal comfort needs.

**FIGURE 1:**
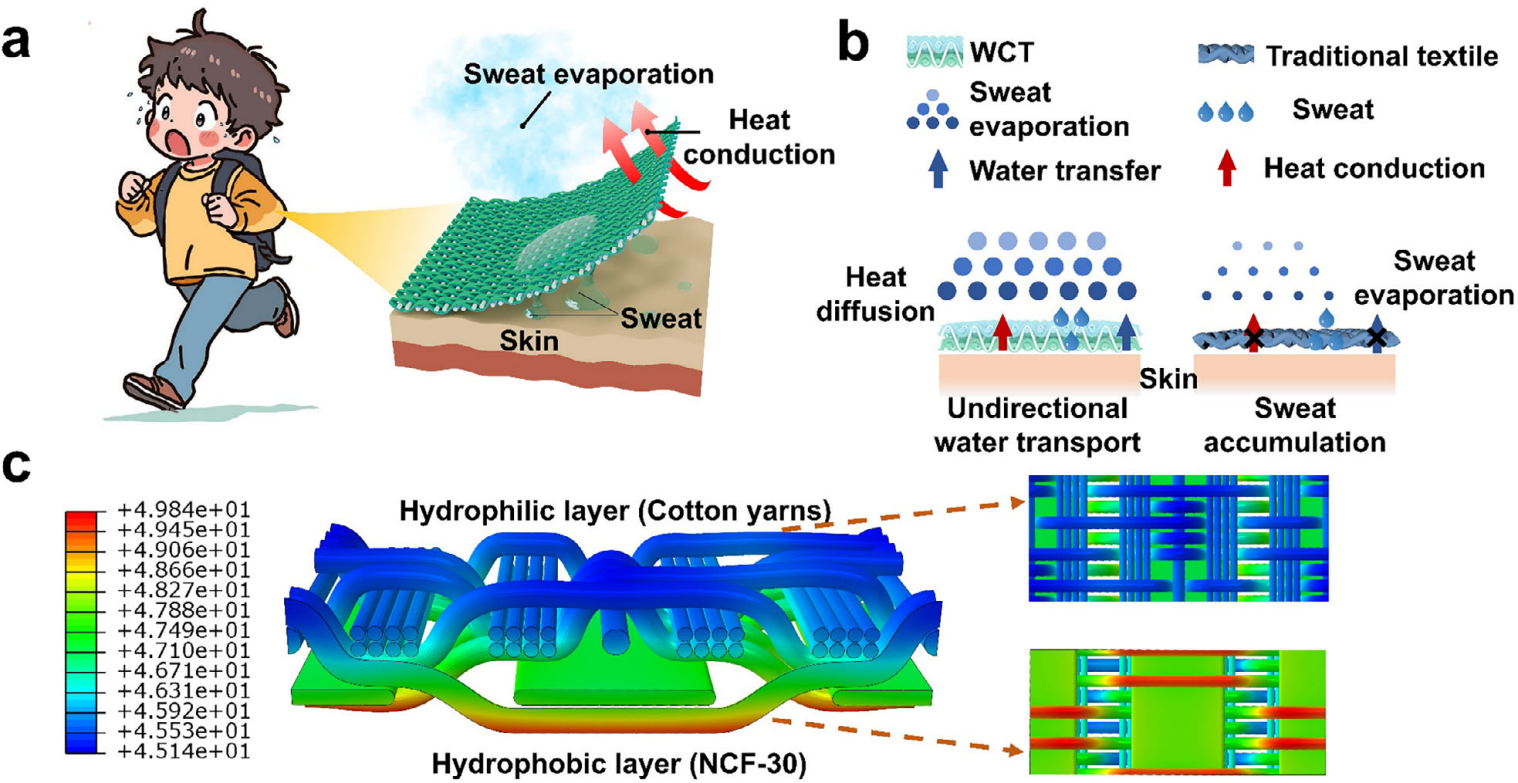
(a) Illustration of evaporative heat dissipation of the WCT. (b)Illustration of directional water transport and heat dissipation in the WCT. (c) Finite element simulation of the heat transfer behavior of the WCT.

Herein, this study innovatively employs biomass‐derived *Juncus effusus* (3DN) as a 3D template to construct an integrated thermally conductive network and fabricate a wearable cooling textile (WCT). The WCT provides thermal conduction and evaporative functions for efficient personal thermohygrosccopic comfort (Figure 1a,b). Boron nitride nanosheets (BNNS) are exfoliated and anchored onto the 3D framework, their 2D structure enables highly efficient thermal transport. To enhance the flexibility and elasticity, the BNNS‐coated 3DN is further impregnated with a thermally conductive solution containing boron nitride and polyurethane (BN/PU). This molecular‐level design ensures effective thermal conductivity by establishing continuous heat transfer pathways while minimizing interfacial thermal resistance. Through combined chemical bonding and physical welding, the BNNS are tightly interconnected along the 3D scaffold, forming a robust and continuous thermally conductive network. As a result, the composite fiber (NCF) achieves a significantly enhanced thermal conductivity (TC = 0.315 W·m^−1^·K^−1^), which facilitates higher heat flow during dynamic thermal transport. We further demonstrate that textile design can direct this heat transfer along desired pathways (Figure 1c). Additionally, a double‐layer weaving technology is employed to fabricate the WCT. The outer layer consists of NCF as a heat diffusion layer, complemented by commercial cotton fiber that serves a moisture‐absorbing inner layer, with polyester fibers incorporated as a cross‐linking support. This *Janus* structure amplifies both the thermal conductivity gradient and the directional water transport capability. During the water diffusion process, the structure generates a Laplace pressure difference, greatly enhancing unidirectional moisture transport (unidirectional water transport index = 476%) and promoting rapid evaporation (water evaporation rate, WER = 5209.92 g/m^2^/day). Owing to its strong thermal conduction and efficient evaporative performance, the WCT offers a promising potential for development of high performance personal cooling materials capable of maintaining thermohygroscopic comfort across diverse daily environments and climatic conditions.

## 2 Results and Discussion

### 2.1 Performance of 3DN and Modified 3DN

As illustrated in Figure 1a, we assumed that the WCT is designed with excellent sweat evaporation and heat dissipation capabilities to achieve thermohygroscopic comfort. Indeed, the heat dissipation layer of the WCT exhibits excellent hydrophobicity due to its micro‐structured surface. In contrast, cotton fibers are hydrophilic and absorb water. Polyester fibers are on the hydrophobic and hydrophilic layer (Figure 1b), creating a wettability gradient that facilitates unidirectional water transport from the inner to the outer layer. This structure allows the WCT to efficiently transfer heat toward the human body, where evaporation is promoted, thereby accelerating cooling. A simplified finite element simulation (COMSOL Multiphysics) was performed to illustrate the underlying heat transfer mechanism, as shown in Figure 1c. The anisotropic TC of WCT arises from the TC gradient across its inner layer (NCF‐30), connected layer (polyester yarns), and outer layer (cotton yarns). Owing to its high TC, the NCF‐30 layer responds rapidly to an external heat resource, displaying a red color up in initial contact with heat flow, and effectively transmits heat to the surrounding regions. Compared to cotton and polyester yarns, NCF‐30 exhibits a more uniform temperature distribution. Consequently, both the experimental and simulated results show a smaller temperature differential between the upper and bottom surfaces of the WCT. These findings confirm the anisotropic TC of the WCT, which significantly enhances heat dissipation and directional heat conduction.

**FIGURE 2:**
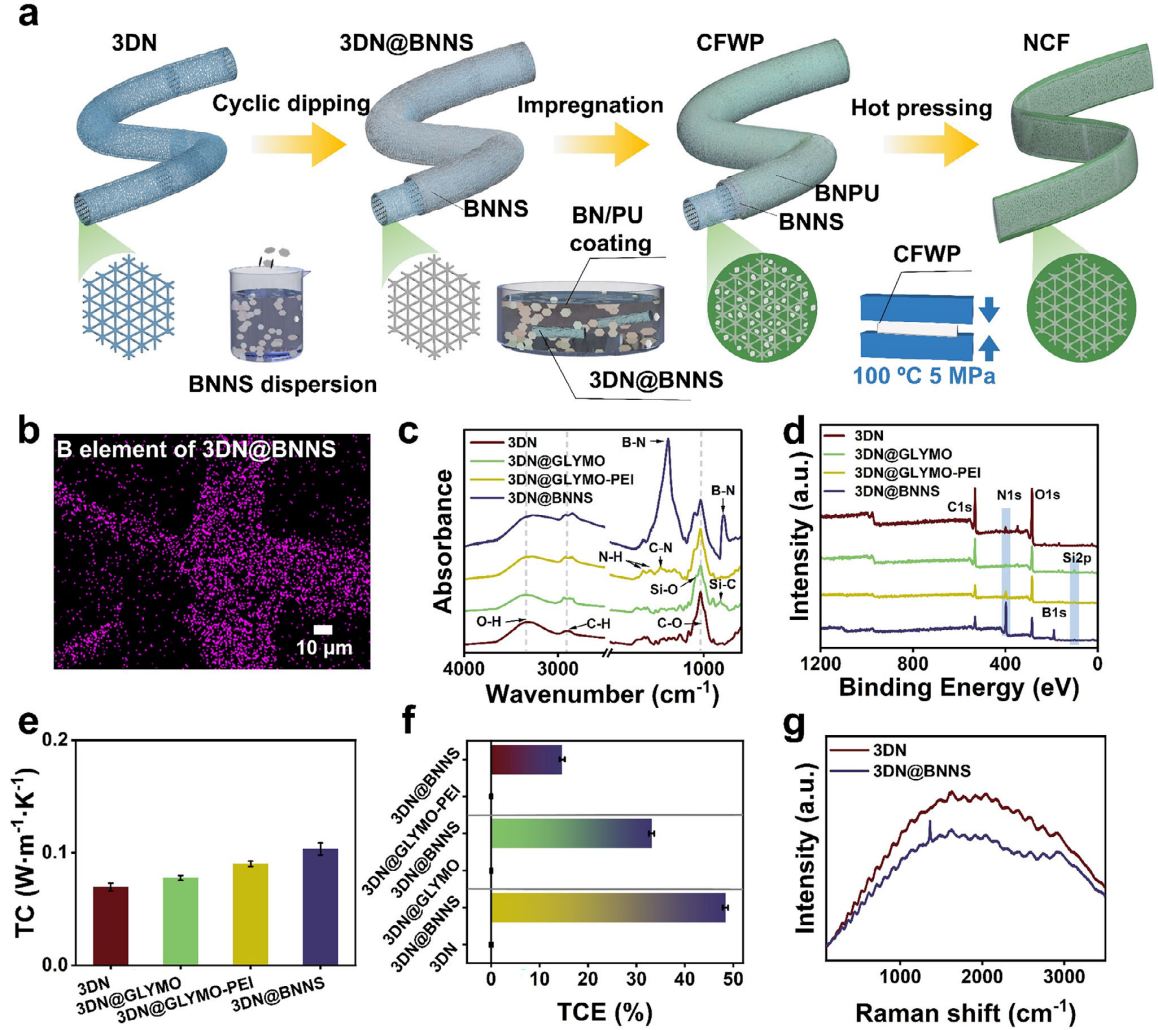
(a) Fabrication of NCF. (b) The B element distribution image of 3DN@BNNS. (c) FTIR spectra. (d) XPS pattern. and (e) TC of 3DN, 3DN@GLYMO, 3DN@GLYMO‐PEI, and 3DN@BNNS fabrics. (f) The thermal conductivity enhancement (TCE) of 3DN@GLYMO, 3DN@GLYMO‐PEI, and 3DN@BNNS fabrics. (g) Raman spectra of 3DN and 3DN@BNNS.

To fabricate textiles with effective cooling performance, several structural requirements must be fulfilled: First, a 3D thermal‐conductive network is essential to enable omnidirectional and rapid heat diffusion for effective heat‐transfer‐based cooling; Second, a *Janus* structure is needed to facilitate the directional absorption and outward diffusion of sweat from the skin. To construct the 3D framework, we initially utilized a biomass‐derived 3D template as the substrate (Figure 2a). More importantly, we developed a method to establish a highly conductive network by directly coating nanosheets onto both the inner and outer surfaces of the 3D network via a combination of chemical modification and physical immersion. Unlike 1D linear or 3D spherical particles, BNNS are 2D, white materials with high TC [28]. Their planar shape facilitates the formation of continuous pathways for heat conduction. This method avoids the mechanical degradation associated with excessive filler loading for improved TC and eliminates the interfacial thermal barrier between nanosheets and polymers during heat transfer. Furthermore, a subsequent hot‐pressing step establishes robust welding between fibers, enabling more efficient multidirectional heat dissipation.

Figure 2a illustrates the preparation of the modified 3DN, which exhibits strong adhesion between BNNS and 3DN, effectively preventing particle detachment (Figure S1). The interconnected 3D microstructure, composed of both orderly arranged BNNS and randomly distributed BN particles, establishes continuous thermal pathways within the composite. BN was exfoliated into nanosheets with fewer layers via ultrasonication, and the resulting BNNS maintained well‐defined morphology and crystal structure, as evidenced by SEM, TEM, FTIR, and XRD (Figures S2–S4) [29, 30]. The BNNS was successfully coated onto the surface of the 3DN, as shown in Figure S5 and Figure 2b. Figure S6 clearly demonstrates full coverage of the 3DN by BNNS, indicating strong interfacial interaction between BNNS and 3DN. Furthermore, FTIR and XPS spectra of the 3DN, 3DN@GLYMO, 3DN@GLYMO‐PEI, and 3DN@BNNS, are shown in Figure 2c,d. New peaks appearing at 821 and 1080 cm^−1^ for 3DN@GLYMO confirm the formation of Si‐C and Si‐O bonds [31]. Additionally, compared with the FTIR spectrum of 3DN@GLYMO, new characteristic peaks at 1574 and 1460 cm^−1^ in 3DN@GLYMO‐PEI correspond to N‐H and C‐N vibrations from PEI [32], indicating successful chemical modification on the surface of the 3DN. As shown in Figure 2d, 3DN@GLYMO exhibits an additional distinct peaks at 102 eV corresponding to Si2p. This suggests that GLYMO was successfully introduced onto the surface of the 3D network structure. The effective introduction of BNNS on the 3DN@GLYMO‐PEI surface is further confirmed by the appearance of a new characteristic B1s peak at 109 eV in 3DN@BNNS. High‐resolution XPS spectra of 3DN@GLYMO, 3DN@GLYMO‐PEI, and 3DN@BNNS show the changes of chemical groups during the modification process in Figures S7–S9 [33]. The TC and TCE of 3DN, 3DN@GLYMO, 3DN@GLYMO‐PEI, and 3DN@BNNS are presented in Figure 2e,f. A significant increase in both TC and TCE confirms the establishement of an effecive themal concuctive pathway. Raman spectra of BN, 3DN and 3DN@BNNS samples in Figure 2g and Figure S10 exhibit a prominent peak near 1368 cm^−1^ for BN, attributed to the high‐frequency E2g mode [34, 35]. A peak shift in 3DN@BNNS suggests chemical interaction between BNNS and the 3DN [36]. These results indicates that PEI and BNNS have been successfully introduced onto the 3DN surface and that the nitrogen functional groups on 3DN have significantly increased after surface chemical modification and layer‐by‐layer self‐assembly treatment. Moreover, the results further verify that BNNS has been successfully introduced into the 3D network skeleton, forming a stable and thermally conductive composite structure.

### 2.2 Performance of NCF

**FIGURE 3:**
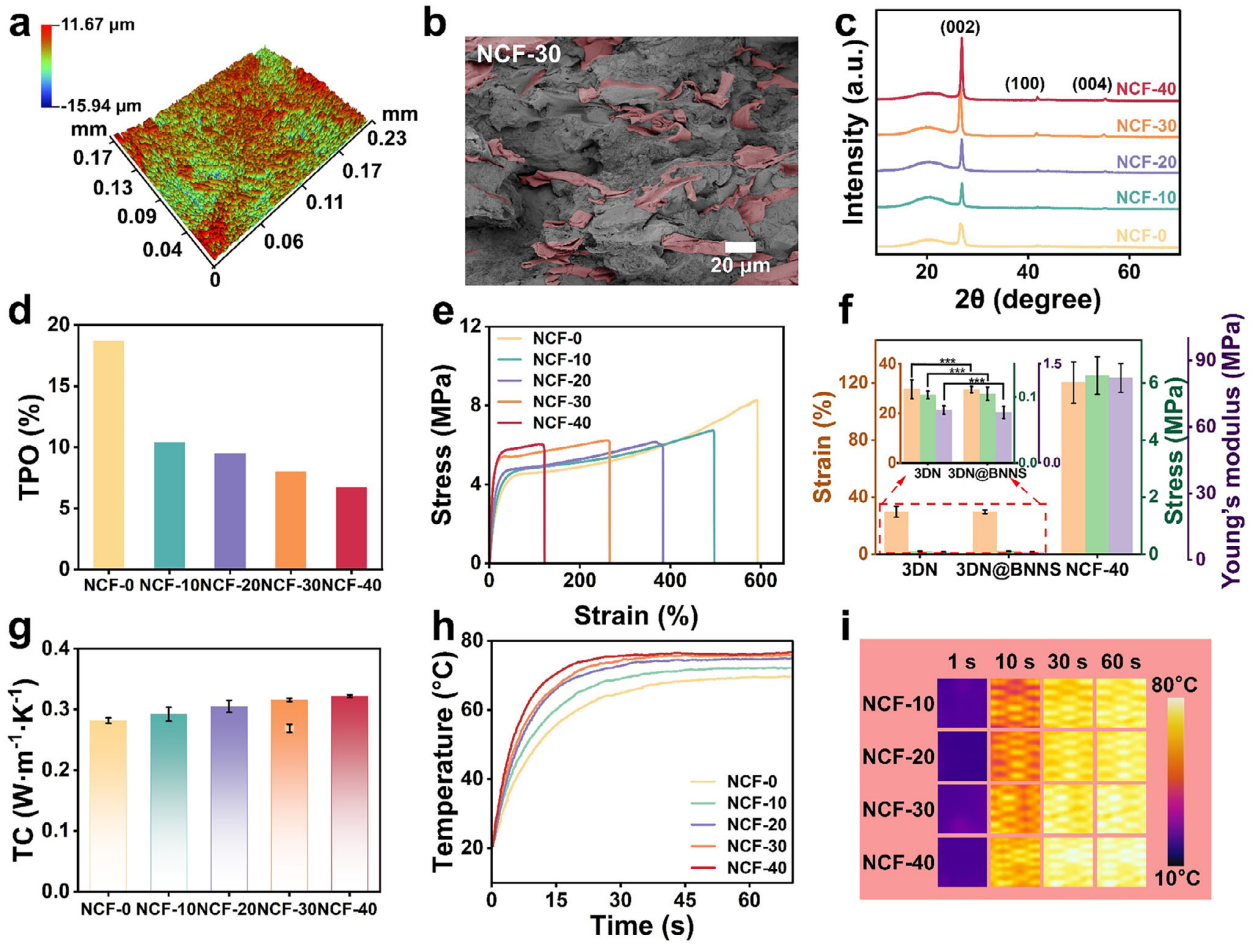
(a) 3D optical profile images of the NCF‐30. (b) Cross‐sectional SEM image of NCF‐30 (The red mark the position of the 3DN@BNNS). (c) XRD pattern of the NCF. (d) Histogram of through‐plane orientation (TPO) calculated from the XRD pattern. (e) Stress–Strain curves of the NCF. (f) Mechanical properties of 3DN, 3DN@BNNS, and NCF‐40. ^*^ *p* < 0.05, ^**^ *p* < 0.01, ^***^ *p* < 0.001, ^****^ *p* < 0.0001. (g) The histogram of TC of NCF fabrics (inner point was the TC of the CFWP fabric). (h) Surface temperature change of NCF during the heating process (from 20°C to 80°C). (i) Infrared thermal images of the NCF plain fabrics.

Furthermore, the inherent mechanical weakness of the 3DN is insufficient for textile applications. To address this, we used flexible BN/PU to wrap 3DN@BNNS, thereby improving both mechanical and TC stability. The surface morphology of 3DN, CFWP, and NCF was examined using 3D optical profilometry in Figure 3a and Figure S11. The surface roughness were 3.11, 3.64, and 2.54 µm, respectively. While the surfaces of 3DN and CFWP appeared heterogeneous, the NCF‐30 exhibited a more homogeneous morphology, which is conducive to uniform water transport. In addition, the inner structure of the NCF was observed by the cross‐sectional SEM images. As indicated in Figure 3b and Figure S12, a continuous 3D thermally conductive network was formed, in which BNNS was uniformly coated on the network in Figure S6. Moreover, BN alignment within this 3D network was observed. The through‐plane orientation (TPO) of BN, calculated from XRD patterns in Figure 3c,d, increased with BN content, confirming that BN tends to align horizontally, benefiting from the 3D thermal conductive pathway and the hot‐pressing process. Mechanical properties of 3DN, modified 3DN, and NCF are shown in Figure 3e,f and Figure S13. Compared with the original 3DN, 3DN@BNNS exhibited a slight improvement in tensile stress, indicating that the modification did not compromise the 3D structure, consistent with SEM images. Due to the reinforcing effect of the BN/PU coating, the NCF composites exhibited significantly enhanced mechanical performance. The tensile stress and strain of NCF‐30 were 7.40 MPa and 245%, which are 6948% and 731% higher than those of 3DN, respectively. Additionally, the Young's modulus of the 3DN, 3DN@BNNS, and NCF‐40 were 0.58, 0.68, and 79.86 mPa, respectively. These results confirm that the integrated 3D network structure combined with the BN/PU coating effectively provides the necessary mechanical strength for practical textile use.

As shown in Figures 2e and 3g and Figure S14, the CFWP fabric exhibited enhanced TC, with increases of 285.81% and 160.04% compared to the 3DN and 3DN@BNNS, respectively. These results indicate that constructing a continuous 3D heat conduction network is crucial for improving the TC of materials even under low filler loading. Figures 2e and 3e, 3g shows the TC growth rate and mechanical properties of 3DN, modified 3DN, and NCF with varying conductive filler content. The TC of the fabrics rose by approximately 16% when the conductive filler content increased from 9.80 to 31.90 wt.%, calculated by the TG shown in Figure S15. However, higher BN content also led to increased aggregation of BN particles and a greater BN‐BN and BN‐PU interfaces, in which in turn reduced the tensile stress and strain of the composite fibers. Considering TC and mechanical properties, we selected the composite fiber with 31.90 wt.% filler (NCF‐30) for further investigation to elucidate its thermal conductivity mechanism. Owing to its high TC, the heat conduction performance of the NCF fabric evaluated, as shown in Figure 3h,i. At 60 s, the surface temperatures of NCF‐0 and NCF‐30 fabrics are 69.52°C and 75.67°C, respectively. Compared to the other samples, the NCF‐30 fabric exhibited superior heat transfer performance, which corresponded to its enhanced cooling performance shown in Figure S16. This result demonstrated the potential of the NCF‐30 fabric for daily thermal management applications.

**FIGURE 4:**
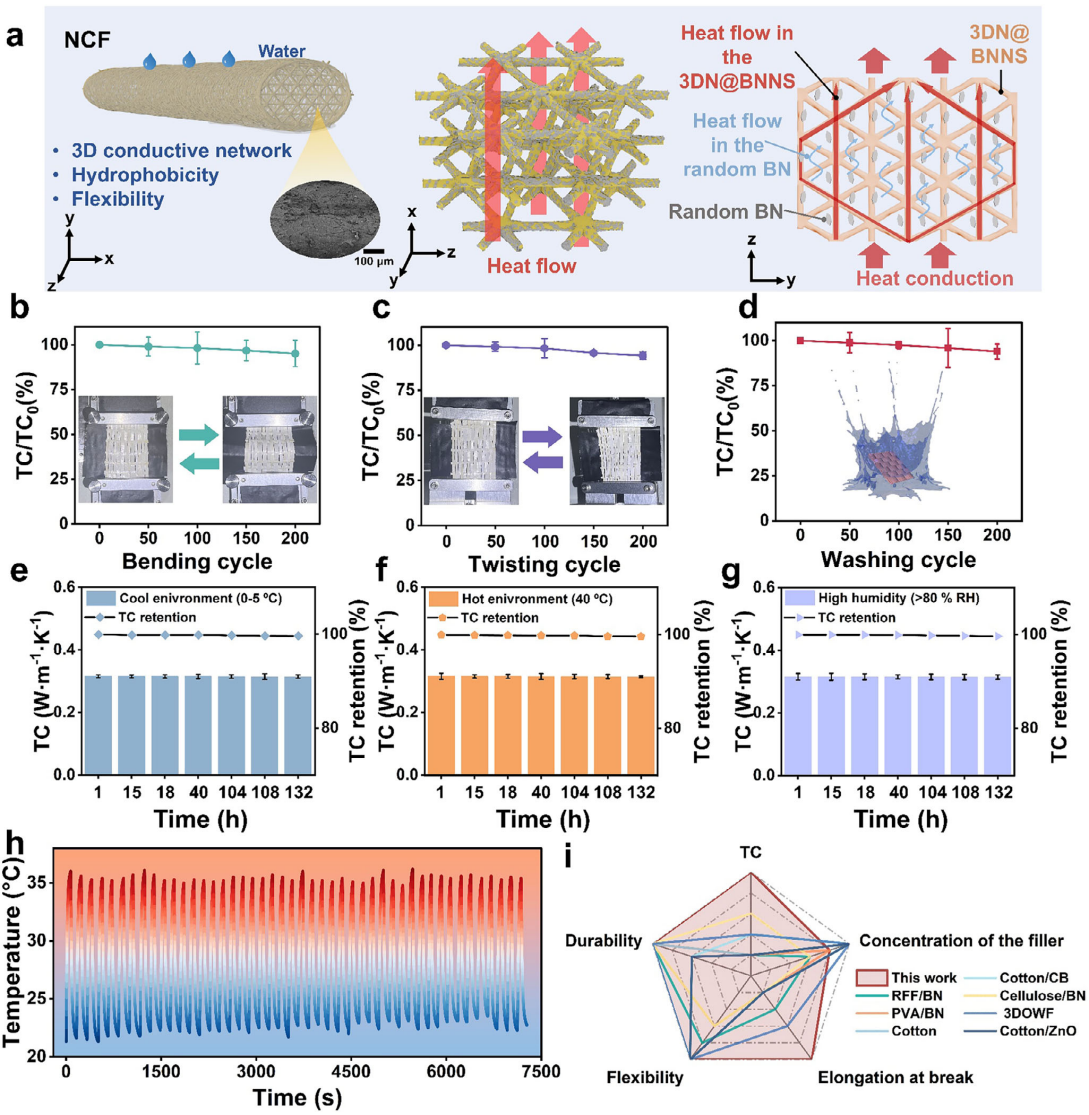
(a) Schematic of the thermal conduction network in the NCF. The durability of the NCF‐30 fabrics: TC after (b) bending, (c) twisting, (d) washing, (e) cooling treatment (0°C–5°C), (f) heating treatment (40°C), and (g) high‐humidity treatment (> 80% relative humidity). (h) Temperature‐response stability during low‐high‐low (20°C–80°C–20°C) thermal cycles. (i) Comparison of key functions between the prepared NCF fabrics and previously reported materials [40, 41, 42, 43, 44, 45].

The microstructure of the NCF confirms the connectivity of BN in the BN/PU and the effective coating of BNNS on the 3D network. Further, the hot‐pressing process promotes the alignment of BN and BNNS, thereby enhancing the through‐plane TC. In addition to establishing a 3D thermally conductive network, hot‐pressing procedure further improves TC by reducing cavities and improving the arrangement of filler. Figure 4a schematically illustrates the heat conduction mechanism in the NCF. First, phonons can be rapidly conducted along the ordered interconnected 3D skeleton. Second, the disordered dispersed BN increases the heat conduction path, which further enhances the anisotropic phonons transport. To investigate the influence of conductive paths on TC, two additional samples were prepared: CFWP and NCF‐0. By combining an aligned 3DN@BNNS framework, a reinforced BN‐PU interface, and randomly distributed BN, the design principles of a naturally ordered structure were applied to the fabric. This led to a significant increase in TC, reaching 0.315 W·m^−1^·K^−1^. The Hashin‐Shtrikman (HS) model was employed to analyze the 3D heat conduction network [37, 38, 39]. The HS model equations are given as Equations (1)–(3):

(1) $$K^{\mathrm{HS}+}=K_{f}\;\frac{2K_{f}+K_{m}-2\varphi_{m}(K_{f}-K_{m})}{2K_{f}+K_{m}+\varphi_{m}(K_{f}-K_{m})}\;$$

(2) $$K^{\mathrm{HS}-}=K_{m}\;\frac{2K_{m}+K_{f}-2\varphi_{f}(K_{m}-K_{f})}{2K_{m}+K_{f}+\varphi_{f}(K_{m}-K_{f})}\;$$

(3) $$X_{\mathrm{interconnectivity}}=\frac{K_{c}-K^{\mathrm{HS}-}}{K^{\mathrm{HS}+}-K^{\mathrm{HS}-}}\;\;$$

where the K^HS+^ (upper boundary) refers to the matrix that is surrounded by filler; K^HS−^ (lower boundary) indicates that the fillers are completely separated by the matrix. K_f_ and K_m_ represent the TC of BNNS and PU or BN/PU, respectively. And φ_m_ and φ_f_ are the weight fraction of the thermal conductivity framework and BNNS. X_interconnectivity_ of BN/PU and NCF‐30 was 7.61 × 10^−15^ and 3.31 × 10^−2^, respectively. Therefore, the interconnectivity of BNNS particles showed excellent interaction due to the 3D heat conduction network. By the construction of a 3D heat conduction network and hot‐pressing procedure, the TC was obviously enhanced. This kind of synergistic effect (*f*) in this binary thermally conductive network system is calculated as Equation (4):

(4) $$f=\frac{\lambda_{AB/M}-\lambda_{M}}{\left(\lambda_{A/M}-\lambda_{M})+(\lambda_{B/M}-\lambda_{M}\right)}\;$$

where 𝜆_AB/M_, 𝜆_M_, 𝜆_A/M_, and 𝜆_B/M_ represent the TC of the binary hybrid system (with the 3D network and the hot‐pressing), the TC of the pure polymer, the composite with only the 3D network, and the composite with only the hot‐pressing process, respectively. The synergistic index f of NCF‐30 is higher than that of NCF‐0, as shown in Figure S17, indicating that both the 3D heat conduction network and the hot‐pressing process play an important role in enhancing the performance of the composite.

The TC stability of the NCF‐30 fabric was examined under mechanical cyclic loading. Figure 4b–d shows the device diagram of the cyclic test. The NCF‐30 fabric exhibited good TC retention, with no significant change observed after 200 bending cycles, 200 twisting cycles, or 200 washing cycles, confirming its structural stability. Furthermore, the fabric exhibited excellent environmental stability under varying conditions, including cold and hot environment, high humidity, and repeated heating‐cooling cycles, which is attributed to its durable structural integrity, as shown in Figure 4e–h. Figure 4i shows a radar plot comparing the performance of NCF‐30 with other reported materials [40, 41, 42, 43, 44, 45]. NCF‐30 shows integrated properties, including a higher TC (0.315 W·m^−1^·K^−1^), a lower filler concentration (31.90 wt.%), a greater elongation at break (245%), as well as good flexibility and durability. These results highlight its strong potential for application in improving body thermal comfort.

### 2.3 Water Transfer Performance

**FIGURE 5:**
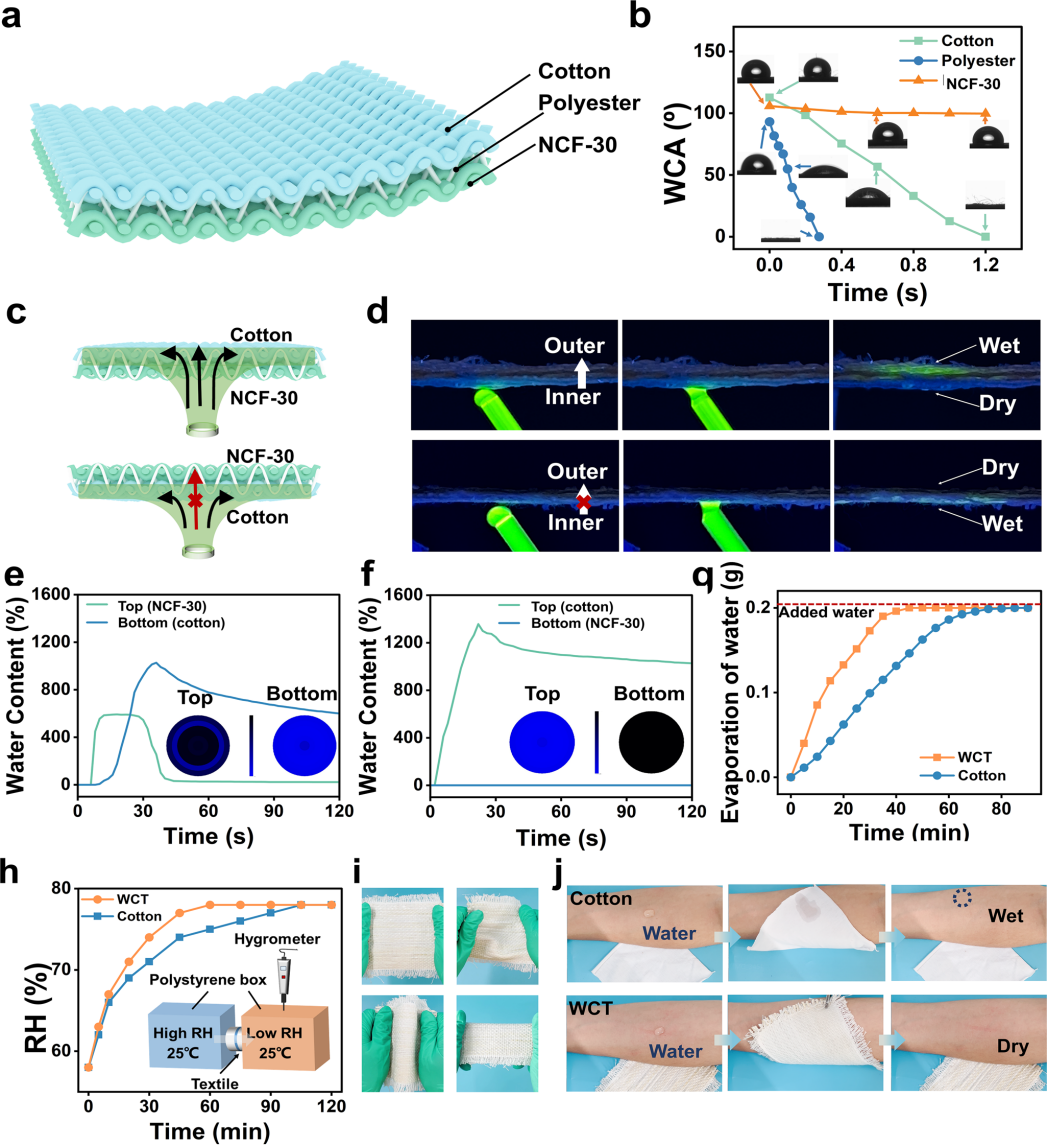
(a) Illustration of the multi‐layer structure of WCT. (b) Water contact angle (WCA) of cotton, polyester, and NCF‐30 within the WCT. (c) Schematic illustration of unidirectional water transport in the WCT. (d) The water droplet moves upward from the inner side to the outer side. The moisture management tester (MMT) results of the WCT when water was dropped on the (e) hydrophobic and (f) hydrophilic layer. The blue and black areas indicated high and low water content, respectively. (g) Water evaporation of the WCT compared to cotton fabric. (h) Relative humidity (RH) equilibrium rate of WCT and cotton fabric. Insert image: schematic of the testing device. (i) Photograph of WCT. (j) Visual comparison of cotton and WCT before and after simulated sweating.

Through structural weaving design, the NCF‐30 fibers formed a hydrophobic layer, cotton constitutes a hydrophilic layer, and polyester connect them, creating a wetting gradient across the textile (Figure 5a; Figure S18). The weaving setup for producing the double‐layer fabric with back warp stitchingconsists of three main components [46, 47, 48]. (i) *Drawing in frame*, a linear drafting method is used with eight heddles, with each warp yarn threaded through one heddle in sequence. (ii) *Reeding Scheme*, one warp thread is drawn through each dent of the reed to ensure even warp distribution. (iii) *Lifting Plan*, the shedding sequence and the heald‐lifting sequence are programmed on a small loom to obtain the required weft‐support structure. The outer layer is woven in a 2/2 basket weave, and the inner layer in a 2/2 twill weave. Due to the different linear densities of NCF‐30 and cotton, eight single cotton yarns were plied together to serve as a one weft yarn. The thicknesses of the WCT, commercial cotton, and polyester fabrics are 1.25 ± 0.02, 0.22 ± 0.01, and 0.41 ± 0.01 mm, respectively. Figures S19 and S20 present optical images of the WCT showing its hydrophobic side, hydrophilic side, and cross‐section, illustrating the multilayer structure design. The plain structures of the commercial cotton and polyester fabrics are shown in Figure S21. This multilayer structure enables unidirectional water transport, allowing mositure to move readily from the skin into the textile for rapid evaporation [49]. The high TC of NCF‐30 promotes rapid transfer excess heat to the fabric surface, thereby accelerating both water evaporationand heat dissipation from the skin. The temperature response of the WCT during heating (50°C and 70°C) is compared with simulation results in Figure S22. These results confirm the fast heat response of the WCT and show only minor difference between experimnetal and simulation data. Wettability of both sides of the WCT was characterized by WCA. WCAs measurements revealed that water droplet remained stable on the NCF‐30 but rapidly penetrated the cotton yarn, owing to its hydrophilicity and capillary pores, and the polyester yarn through capollary action (Figure 5b; Figure S23) Water droplets spread on the cotton side within 0.6 s, while the NCF‐30 side remained relatively dry (Movie S1). When the NCF‐30 side was faced upward, water droplets required at least 10 s to penetrate (Movie S2). The gradient in wettability facilitates directional water transport.

In practical applications, the hydrophobic layer is used as the inner side in contact with the skin, while the hydrophilic layer is exposed to the environment to wick excess sweat away from the skin and enhance evaporation. To visualize unidirectional transport, a schematic of antigravity water movement in the WCT and an optical image of fluorescent droplet (0.1 wt.%) transport are shown in Figure 5c,d, respectively. When fluorescent droplets (20 µL) contact hydrophobic layer, they are rapidly transported from the hydrophobic side(dark blue area) to the hydrophilic side (bright green area). Conversely, when fluorescent droplets are applied to the hydrophilic side, they are blocked by the hydrophobic side and spread only laterally on the hydrophilic surface (Movies S1 and S2). These observations demonstrate that the wetting gradient design endows the WCT with effective unidirectional water transport capability.

The directional water transport capacity of the WCT was further evaluated by a MMT (Figure S24). Figure 5e,f shows the changes in relative water content on both sides of the WCT. When an artificial sweat (saline solution) was added to the hydrophobic layer (top), its water content initially increased and then gradually transferred to the hydrophilic layer (bottom), as shown in Figure 5e. This directional transport indicates that the skin‐side surface could remain dry. In contrast, when the artifical sweat was applied to the hydrophilic layer (top), it was retained there, which prevented it from penetrating into the underlying hyrophobic layer (bottom). This behavior is attributed to the wettability gradient across the fabric and the capillary action of the polyester in the hydrophilic layer, which draws artifical sweat from the hydrophobic layer. The directional water transport index (R) of WCT from the hydrophobic side to the hydrophilic side was 476% and 1031% in the reverse direction. A schematic of the directional perspiration mechanism is illustrated in Figure S25. These results confirm the excellent unidirectional water transport capacity of WCT.

In addition to the unidirectional water transport capability, the WER and moisture permeability are also critical for practical applications. The water evaporation of the WCT was 0.267 g, which is nearly twice that of cotton (0.142 g) in Figure 5g. Compared with conventional cotton textiles, the WCT placed between two transparent boxes with different RH reached RH equilibrium more rapidly at 25°C (Figure 5h). These results indicated that the WCT possesses a high WER and excellent water vapor permeability, which can be ascribed to its wettiability‐gradient structure and efficient unidirectional water transpor.

Figure 5j and Figure S26 show a photograph of WCT, cotton, and polyester fabric before and after simulated sweating. Skin covered with the WCT dried quickly and remained comfortable within seconds due to spontaneous directional sweat transport. In contrast, cotton and polyester retained considerable moisture at the skin‐contact surface.

### 2.4 Evaporative Cooling Performance

**FIGURE 6:**
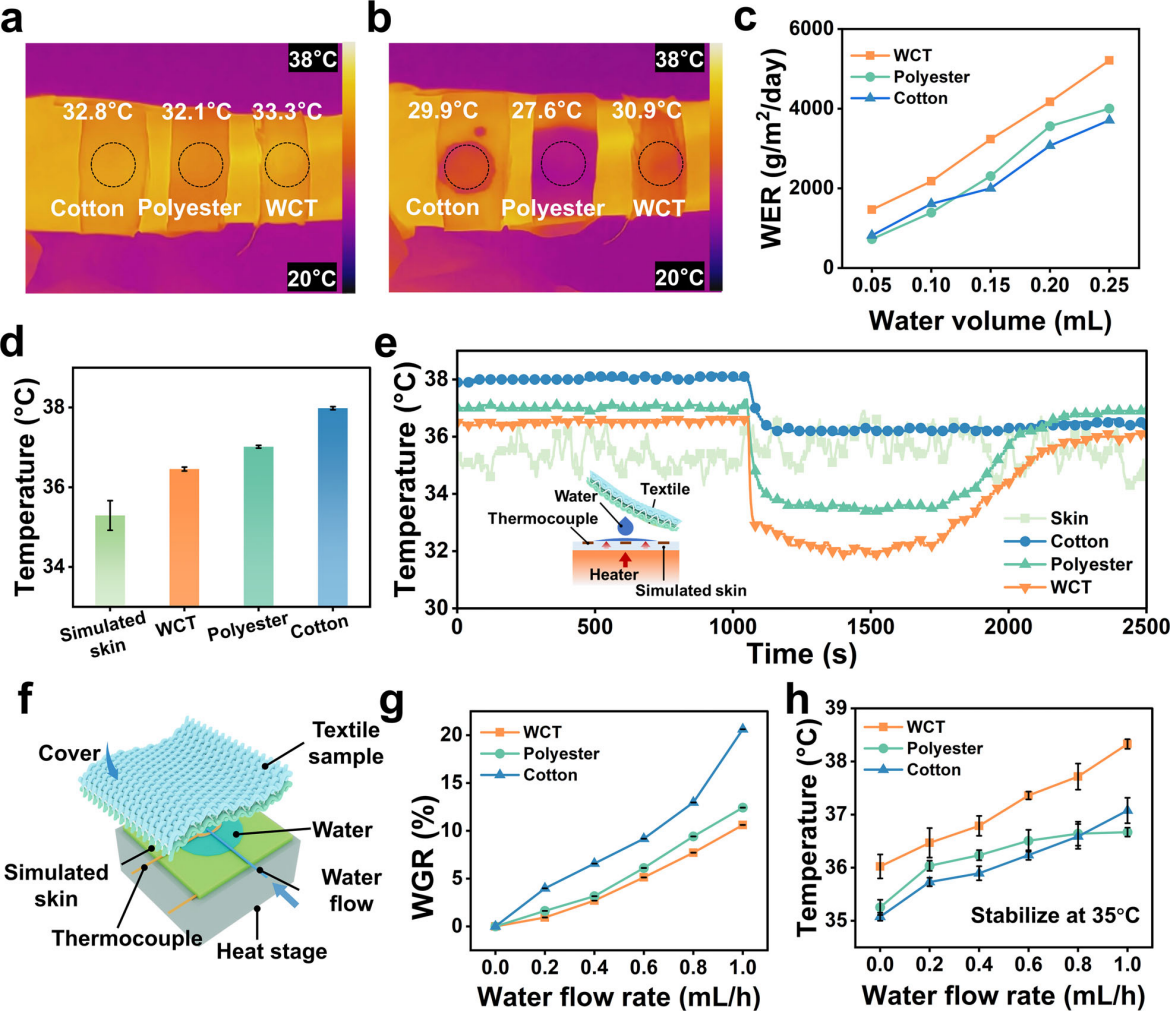
Infrared thermal images of cotton, polyester, and WCT under (a) dry and (b) wet conditions. (c) WER of cotton, polyester, and WCT vs. water volume at a constant heating temperature of 38°C. (d) Average surface temperature of the simulated skin after drying textile covering. (e) Temperature difference between cotton‐covered, polyester‐covered, and WCT‐covered skin simulators under dry and wet conditions. (f) Schematic of the steady‐state water evaporation testing device. (g) The water gain ratio (WGR) of textile samples under varying water flow rates. (h) Heating temperature of the simulated skin at different water flow rates.

To evaluate the evaporation performance of WCT, cotton, as the most widely used textile, was chosen as the primary textile for comparison. Polyester, known for its moisture‐wicking and quick‐drying properties, was also used for comparison. Figure 6a,b shows infrared thermal images of cotton, polyester, and WCT covering the arms of volunteers under dry and wet conditions, respectively. The surface temperatures of the WCT were 33.3°C and 30.9°C in dry and wet conditions, both higher than those of cotton and polyester, indicating its good TC in both dry and wet states. High TC facilitates water evaporation on the textile surface and more rapid dissipation of excess heat from the skin, thereby promoting body thermal comfort. Water evaporation is an effective way for the body to cool, and the water evaporation capacities of the WCT with those of conventional textiles were compared. Different volumes of water were added to a constant‐temperature heating stage (38°C), and the textile sample was covered. Figure 6c shows the relationship between the WER and the amount of water. For all three textiles, the WER increased with the added water volume. At 38°C, the WER of the WCT was significantly higher than those of cotton and polyester, indicating that the synergy between its unidirectional water transport and high thermal conduction provides a clear advantage in accelerating water evaporation. Transient water evaporation tests were further conducted to characterize the cooling capacity of the WCT. Uder dry conditions, the average temperature of the simulated skin covered by the WCT was 36.50°C, which is lower than that of the polyester (37°C) and cotton (38°C) (Figure 6d). After the simulated skin temperature stabilized, 0.2 mL of water at ambient temperature was injected onto its surface to simulate the evaporation of water after human sweating (Figure 6e). The temperature of the simulated skin dropped sharply because of heat dissipation and evaporative heat transfer, where the average temperature of the simulated skin covered by the WCT in the wet state was 32.1°C. The WCT‐covered simulated skin temperature during evaporation was lower than that under conventional textiles, with an average reduction of 4.1°C compared to cotton. These results demonstrate that wearing the WCT promotes fast sweat evaporation and maintains a lower skin temperature. Therefore, the WCT exhibits good TC under both dry and humid conditions and demonstrates effective active‐cooling performance. To further clarify its superior evaporation and cooling properties, a steady‐state evaporation test was conducted. This test better distinguishes the evaporation and cooling behaviors of textiles under conditions resembling human sweating. The measurement device is illustrated in Figure 6f. The WGR of the WCT was consistently lower than those of cotton and polyester, and water on the WCT evaporated faster at the same water flow rate. Compared with cotton, the WGR of the WCT was reduced by 94% at a water flow rate of 1.0 mL/h. During simulated heavy sweating, the WCT remained relatively dry compared to cotton.

Figure 6g,h shows the relation between heating temperature and water flow rate. Under dry conditions, the heating temperature of WCT was higher than those of cotton and polyester, indicating its outstanding heat dissipation capacity. Moreover, the heating temperature of the WCT remained higher than those of conventional textiles, and the heating temperature is 1.2°C higher than that of cotton at a water flow rate of 1.0 mL/h, indicating that the WCT releases more heat and exhibits stronger cooling at a given water flow rate. Therefore, the WCT exhibits clear advantages in both TC and sweat evaporation cooling efficiency, highlighting its strong potential for next‐generation personal‐cooling textiles.

## 3 Conclusion

We fabricated WCT using a 3D cladding strategy of BNNS for effective human thermal management under various environmental conditions. The 3D thermal conductive pathway formed by the direct modification of BNNS on a natural network scaffold to fabricate highly thermally conductive composite fibers (NCF‐30), which were then woven into a Janus‐structured textiles using a back warp stitching double configuration. Due to the high TC of BN, the multilevel structure of the WCT meets the requirements of the human body. The continuous 3D thermal conduction network endowed NCF‐30 with a high TC of 0.315 W·m^−1^·K^−1^. Combined with the excellent unidirectional water transport capacity of the WCT (476%) and high WER (5209.92 g/m^2^/day), the synergistic effect of efficient heat conduction and rapid water evaporation accelerated body heat dissipation. In simulated skin tests, the WCT kept the surface 1.5 and 4.1°C cooler than cotton under dry and wet conditions, respectively. During steady‐state evaporation test, the WGR of the WCT was reduced by 94%, compared with the cotton fabric, while the simulated skin heating temperature increased by 1.2°C, confirming its superior water evaporation speed and heat dissipation capacity. These results demonstrate that the WCT exhibits high TC and excellent directional water transport, underscoring its broad potential for improving human thermal comfort in practical applications.

## Conflicts of Interest

The authors declare no conflict of interest.

## Supporting information




**Supporting File 1**: advs73769‐sup‐0001‐SuppMat.docx.

**Supporting File 2**: advs73769‐sup‐0002‐MovieS1.mp4.

**Supporting File 3**: advs73769‐sup‐0003‐MovieS2.mp4.

## Data Availability

The data that support the findings of this study are available from the corresponding author upon reasonable request.
