# Supplementary material for: Wearable Cooling Textiles of Thermal Conduction and Sweat Transfer for Personal Thermal Management
Source: Adv Sci (Weinh). 2026 Jan 26;13(16):e23061. doi: 10.1002/advs.202523061 (PMC13042882; doi:10.1002/advs.202523061)
Supplement: Supplementary file 1 — Supporting File 1: advs73769‐sup‐0001‐SuppMat.docx. [file ADVS-13-e23061-s002.docx]

Supporting Information

**Wearable Cooling Textiles of Thermal Conduction and Sweat Transfer for Personal Thermal Management**

Jiajing Zhang, Jiahao Xu, Chunhua Zhang*, Liangjun Xia*, Xin Liu, and Weilin Xu

State Key Laboratory of New Textile Materials and Advanced Processing, Wuhan Textile University, Wuhan 430200, People’s Republic of China.

E-mail: chzhang@wtu.edu.cn (Chunhua Zhang); liangjun_xia@wtu.edu.cn (Liangjun Xia)

Jiajing Zhang

College of Textile Science and Engineering, Zhejiang Sci-Tech University,

Hangzhou 310018, People’s Republic of China.

**Supplementary Information contains:**

Supplementary Experimental Section

Supplementary Figures S1-S26

Supplementary Movies S1-S2

1. Supplementary Experimental Section

1.1 Materials

Juncus effusus (3DN) was provided by Jiangxi Juncus effusus Co., Ltd. (China). Hexagonal boron nitride (BN, average diameter : 1-2 μm), polyethyleneimine (PEI, 98 %, Mw=800), and 3-glycidoxypropyltrimethoxysilane (GLYMO) were provided by Macklin Biochemical Technologies Co., Ltd. (China). *N, N*-Dimethylformamide (DMF), toluene (TOL), and isopropyl alcohol (IPA) were provided by Sinopharm Chemical Reagent Co., Ltd. (China). Polyurethane 1185A (PU) was supplied by BASF Co., Ltd. (Germany). Polyester yarn (40S/2), polyester fabric, cotton yarn (40S/2), and cotton fabric were purchased from local suppliers.

1.2 Experimental Section

*Fabrication of WCT*: The preparation process of wearable cooling textile (WCT) includes three main steps: modification of the three-dimensional network (3DN), fabrication of the 3DN-cooling fibers (NCF), and assembly of the NCF.

*Modification of 3DN*: First, 2 wt% of BN was dispersed in a mixed solvent of IPA and H_2_O (mass ratio of 9:1) and ultrasonicated for 6 min at 50 Hz. The supernatant was then collected and dried in an oven at 100 °C for 12 h, yielding BNNS. Next, 3 wt% 3DN and GLYMO (1:2 mass ratio) were added to water and stirred for 2 h. PEI was then added to the mixture and stirred for 2 h. The modified 3DN was finally dried at 110 °C for 30 min. The samples modified with GLYMO alone and with both GLYMO and PEI were designated 3DN@GLYMO and 3DN@GLYMO-PEI, respectively.

*Preparation of NCF*: A suspension was prepared by dispersing 1.25 wt% % BNNS in a mixed solvent of IPA and water (1:1 weight ratio). A separate aqueous solution containing 0.12 wt% PEI was also prepared. The 3DN@GLYMO-PEI was then sequentially immersed in these two solutions to obtain 3DN@BNNS. Subsequently, 15 wt% BN and PU at varying mass ratios of 0:10, 1:9, 3:7, and 4:6 were dissolved in a mixture of DMF and TOL (1:1 mass ratio) and stirred at 500 rpm for 3h to obtain the BN/PU solution. The 3DN@BNNS was immersed in the obtained solution and placed under vacuum to remove bubbles. The primary fibers were then transferred to a water coagulation bath for 2 h and dried at 80 °C for 2 h, yielding CFWP. Finally, the dried CFWP was hot-pressed at 100 °C and 5 MPa for 2 min to obtain the NCF. Based on the BN/PU mass ratio in the solution, the resulting fibers were labeled NCF‑0, NCF‑10, NCF‑20, NCF‑30, and NCF‑40, corresponding to the ratios 0:10, 1:9, 3:7, and 4:6, respectively.

*Fabrication of WCT*: Large-scale production of the WCT was carried out on an automatic textile loom (Model SGA, manufactured by Ning Bo Textile Machinery Co., Ltd.) using a back warp stitching double configuration. The WCT comprises a system of warp yarns and two sets of weft yarns. The warp yarns were separated into upper and lower layers using different weft yarns (cotton yarns and NCF-30). The inner layer was woven in a 2/2 twill weave, while the back layer featured a 2/2 basket weave. Eight heddles were used in sequence for thresding, achieving a density of 60 threads per 10 cm. Each steel reed accommodates one thread with unidirectional tension control during the stretching stage. The weaving thread sequence was set as one inner yarn, two back yarns, and one inner yarn to produce WCT.

1.3 Characterizations

The micro-morphology of NCF was observed using a scanning electron microscope (SEM, JSM-7800F, Japan). AElement distribution was analyze by the energy dispersive X-ray spectroscopy (EDX, X-MaxN, Britain). Surface morphology of 3DN, CFWP, and NCF was assessed with an optical profilometry to determine microscale roughness. The measurements were evaluated by a 3D optical surface metrology system (Bruker Contour GT-K 3D, Germany). Functional groups were characterized using Fourier transform infrared spectrometer (FTIR, Nicolet iS50, America) in the wavenumber range of 400 to 4000 cm^-1^. The hydrophilic properties were evaluated using a contact angle measurements (OCA15EC, Dataphysics, Germany) with 5 μL H_2_O droplets. Tensile tests were conducted on an electronic universal material tester (Instron 5943, America) at a speed of 100 mm/min and an initial gauge length of 10 mm followed by GB/T 14344-2022 standard. In addition, the data was indeed analysed by the soft IBM SPSS27, as SPSS provides more comprehensive information, facilitates easier interpretation of results, and enhances the level of accuracy. During the data analysis, we adopted the one-way ANOVA test method to verify the significance testing of data. The P-value refers to the probability that the difference between the two being compared is caused by opportunity. The smaller the P-value is, the more reason there is to believe that there are differences between the compared data (*P < 0.05, **P < 0.01, ***P < 0.001, ****P < 0.0001.). X-ray diffraction analysis was conducted on an X-ray diffractometer (XRD, Empyrean, Netherlands) using Cu Kα radiation at 40 kV and 40 mA. The elements of the sample were tested by X-ray electron spectrometer (XPS, Shimadzu axis supra+, Japan). The exit angle and test depth were 45 ^◦^ and 10 nm from the surface, respectively. All the tests were performed at a temperature of 26 ºC and a humidity of 60 %.

1.4 The thermal property test

The thermal property was tested by a thermogravimetric analyzer (TG, STA 2500 Regulus, Germany) under N_2_ atmosphere at 10 °C/min. The thermal conductivity (TC) was measured using a transient plane thermal conductivity meter (DRPL-II, Xiangtan Xiangyi Instrument Co., Ltd., China). The instruments used for evaluating heat response performance included an infrared thermal imager (FLIR, E8-XT, Teledyne FLIR, America), a heat platform (4030, Bangyuan Instrument Co., Ltd., China), thermocouple temperature sensor (TA612C, TASi Instrument Co., Ltd., China) to record the surface temperature change.

1.5 The moisture management test

The water evaporation ratio (WER) was determined according to GB/T 21655.1 standard. Water (0.2 g) was dropped into the sample (100×100 mm), and the weight change was recorded over time. Two closed polystyrene boxes were used for the moisture permeability test, and textiles were fixed between them. Firstly, two polystyrene boxes were filled with a humidifier and a dehumidifier to adjust the humidity to 58 % and 90 %, respectively, and the temperature was maintained at 25 °C. Then, the sample was placed between two boxes (the hydrophobic layer of the WCT was close to the high-humidity box), the ventilation was opened, and the humidity inside the box was recorded.

*Transient evaporation test*: A silicone plate (OUPLI, 5×5×0.3 cm, China) was placed on a heating platform maintained at 38 °C to simulate skin temperature. The surface temperature of the simulated skin covered with the fabric was monitored in real-time using a thermocouple (k-type, AIDIWEN, China) connected to a digital thermometer. The transient WER was calculated from the evaporated water weight and evaporation time.

*Steady-state evaporation test*: Steady-state evaporation tests were performed on a simulated skin sepup, where a silicone tube was fixed onto the simulated skin surface, and the water in the tube was supplied to the simulated skin at a constant flow rate. The simulated skin surface was covered with the fabric. The simulated skin temperature fluctuated around 35 °C by adjusting the heating platform temperature. After reaching a steady state, the weight of the fabric was measured and the temperature of the heating platform was recorded. The water gain ratio refers to the percentage of water absorbed by the dry sample mass. All tests were performed under controlled amibient conditions of 26±2 °C and 60±10 % relative humidity.

1.6 Quantitative calculation of the directional water transport capacity

The directional water transport capacity was assessed using a moisture management tester (MMT, M290, SDLATLAS, Ltd., China) in accordance with GB/T 21 655.2-2019 regulations. The sample was placed horizontally between paired resistance sensors, and the top surface was then wetted with saline solution (0.21 g, 0.9 wt% sodium chloride in water). The associated electrical resistance was used to quantify the relative moisture content of the top and bottom surfaces. The following equation illustrates how the one-way transport index (R) was calculated by dividing the total water content difference between the top and bottom surfaces by the test time:$\begin{aligned} \text{R=}\frac{\text{1}}{\text{T}}\int\left[ \text{U}_{\text{b}}\text{(T)-}\text{U}_{\text{t}}\text{(T)} \right]\text{dT}\#\left( 1 \right) \end{aligned}$

Where Ut and Ub are the moisture contents of the top and bottom layers, and T is the testing period, respectively. Liquid diffusivity was measured by dropping 20 μL of blue ink on the front and back of the textiles and recording with a camera.

1.7 Durability test

To evaluate the durability of WCT, a series of durability tests were performed, including bending, twisting, and washing cycles Mechanical durability was assessed using a flexible electronic tester (FT2000, Shanghai Mifang Electronic Technology Co., Ltd, Shanghai, China). The textile was clamped on the tester and subjected to repeated bending of 90 degrees and twisting of 60 degrees. TC after bending (TC_b_) and after twisting (TC_w_) was then measured to evaluate performance retention. Washi fastness was evaluated by agitating samples in water at 100 rpm. After washing, the sample was drieen at 100 °C, and TC after washing was determined (TC_h_). Environmental resistance tests was also performed. WCT samples were exposed to cold environment (0-5 ºC), hot environment (40-45 ºC), and high-humidity (over 80 %) for defined durations. TC was subsequently measured after cold (TC_c_), hot (TC_o_), and high-humidity (TC_u_) exposure. TC retention were calculated as (TC_b_ or TC_w_ or TC_h_ or TC_c_ or TC_o_ or TC_u_/TC)*100%.


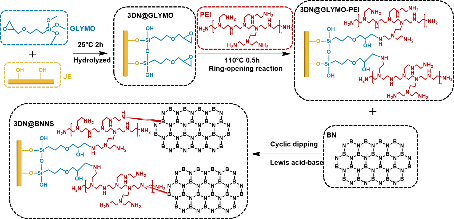


**Figure S1.** The diagram of the reaction mechanism for 3DN@BNNS.


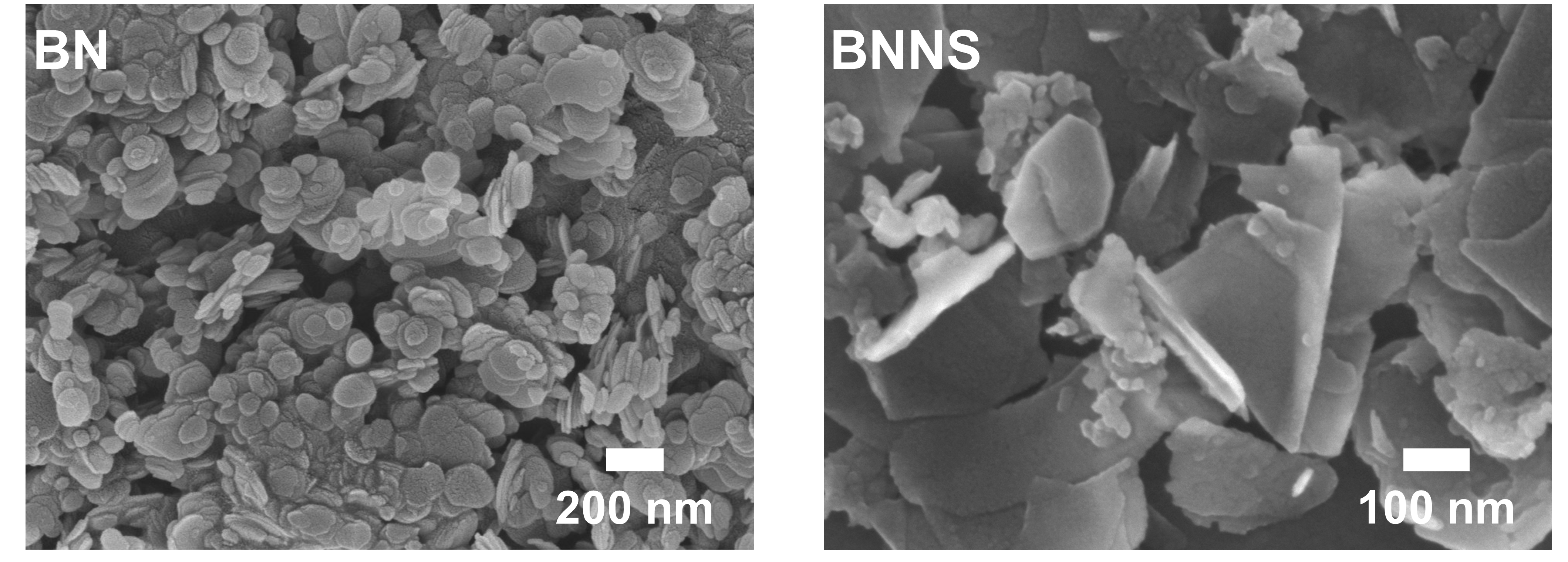


**Figure S2.** SEM images of BN and BNNS.


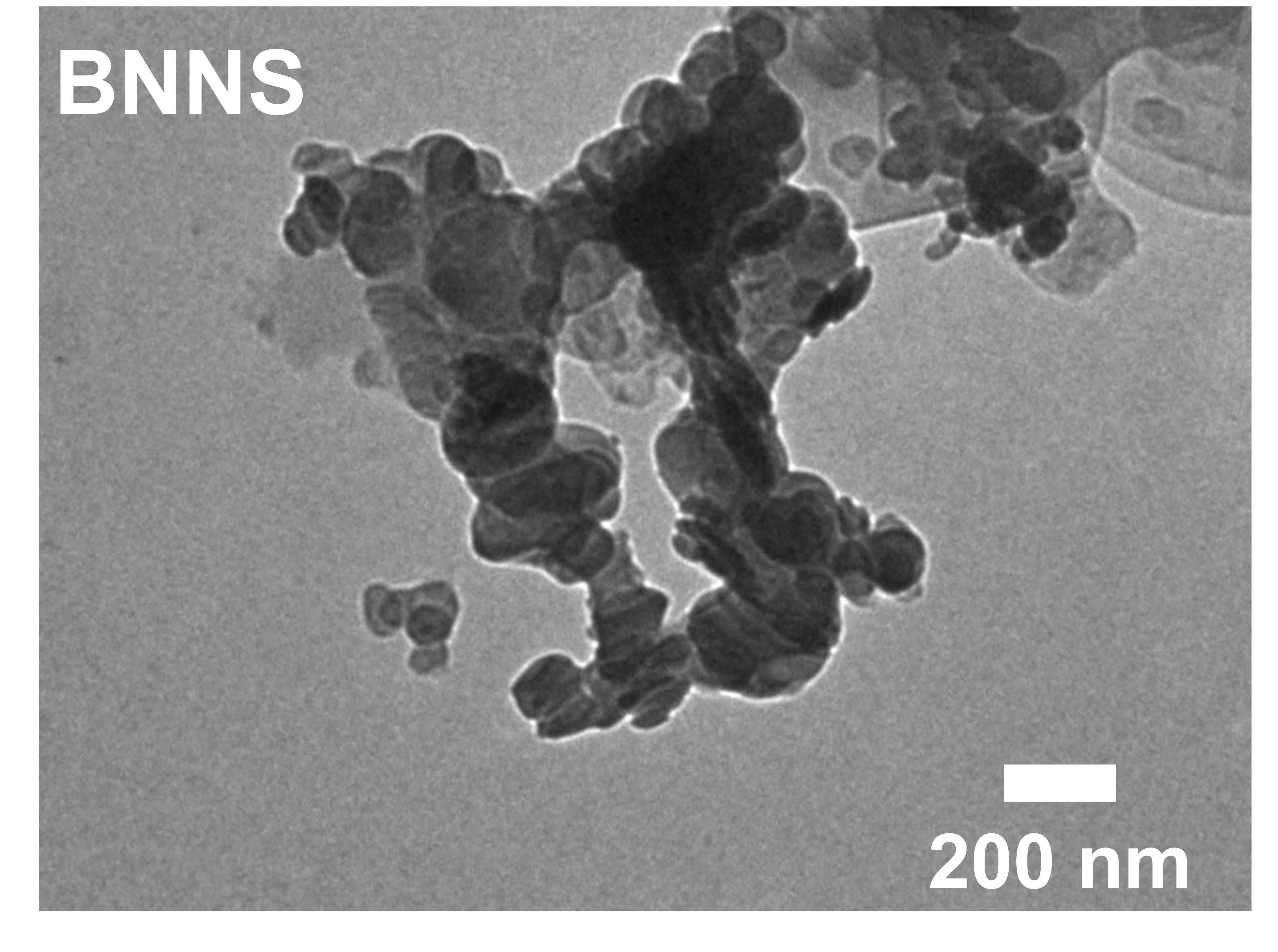


**Figure S3.** TEM image of BNNS.


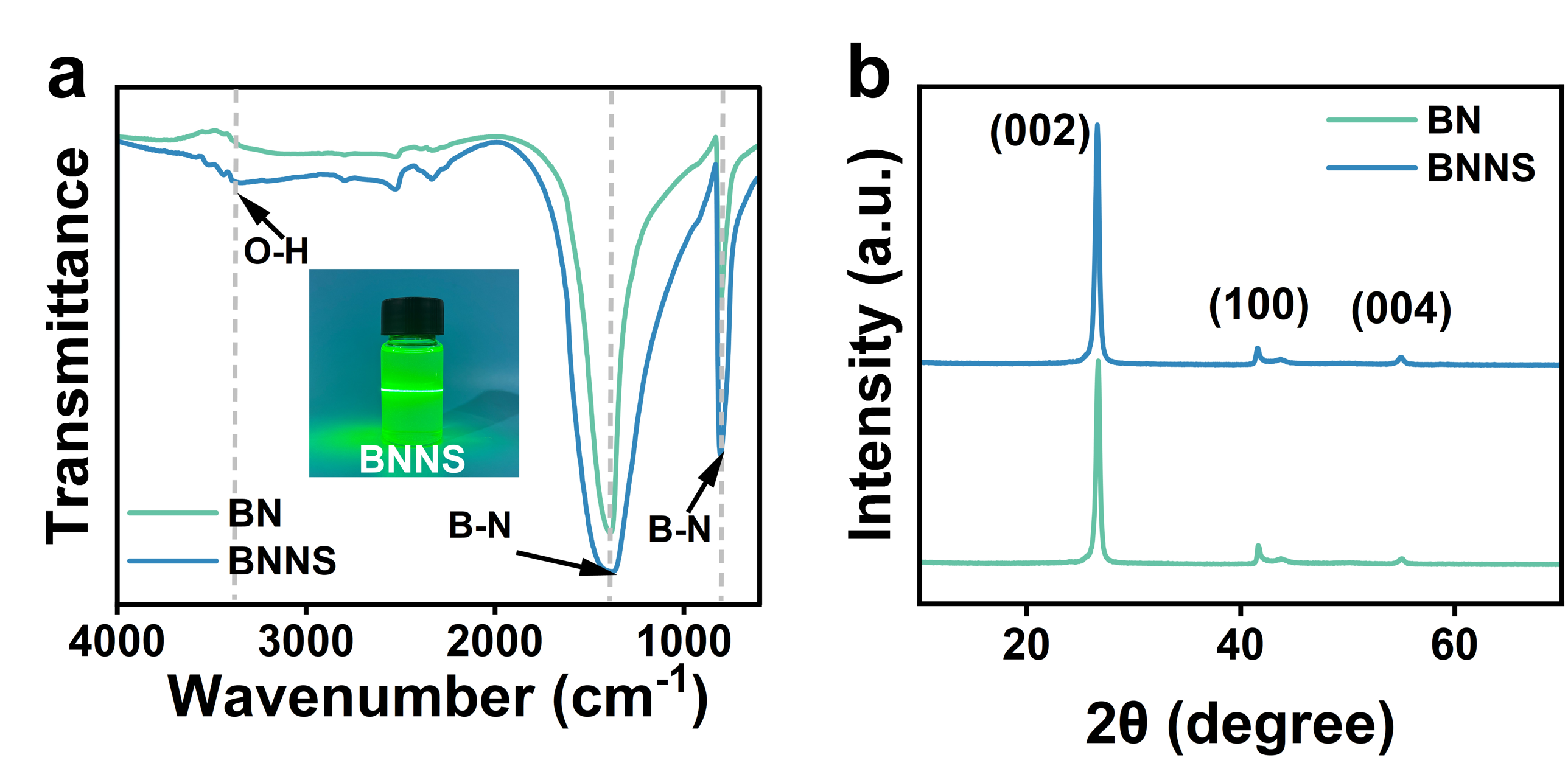


**Figure S4.** (a) FTIR spectra and (b) XRD patterns of BN and BNNS.


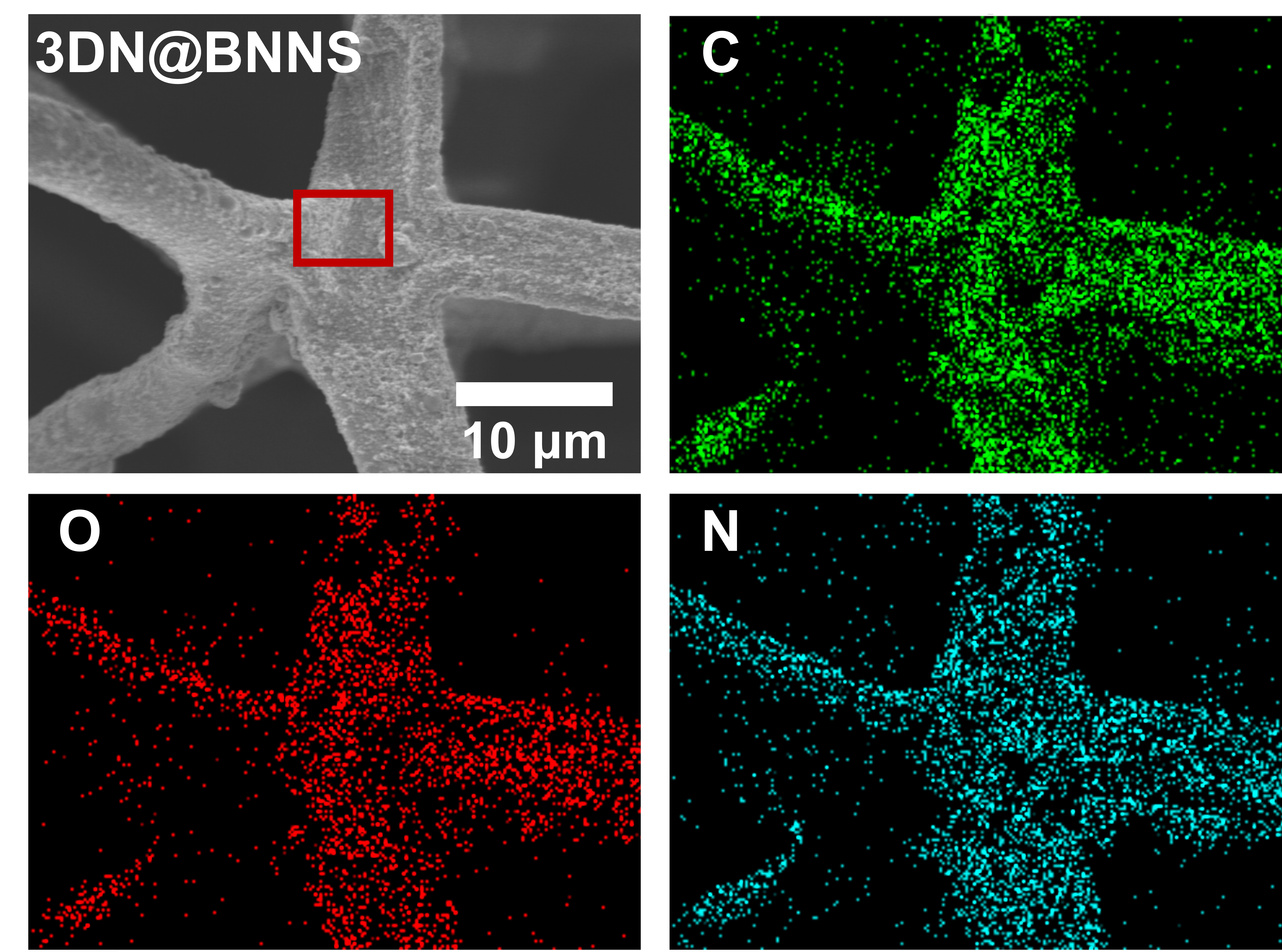


**Figure S5.** SEM and C, O, N element distribution images of 3DN@BNNS.


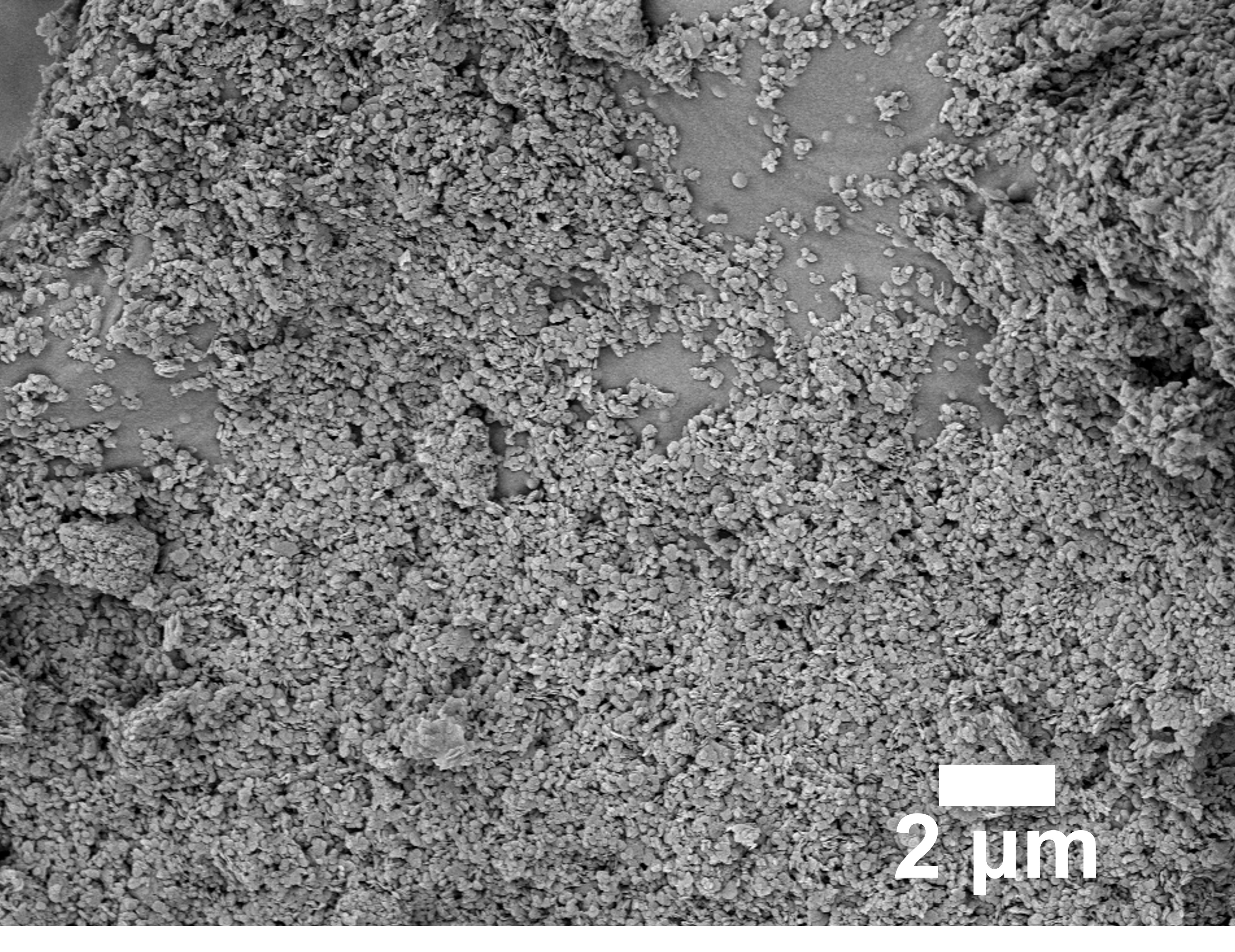


**Figure S6.** SEM image of 3DN@BNNS.





**Figure S7.** High-resolution XPS spectra of C1s, O1s, and Si2p of 3DN@GLYMO.





**Figure S8.** High-resolution XPS spectra of O1s, N1s, and Si2p of 3DN@GLYMO-PEI.





**Figure S9.** High-resolution XPS spectra of O1s, N1s, and B2p of 3DN@BNNS.


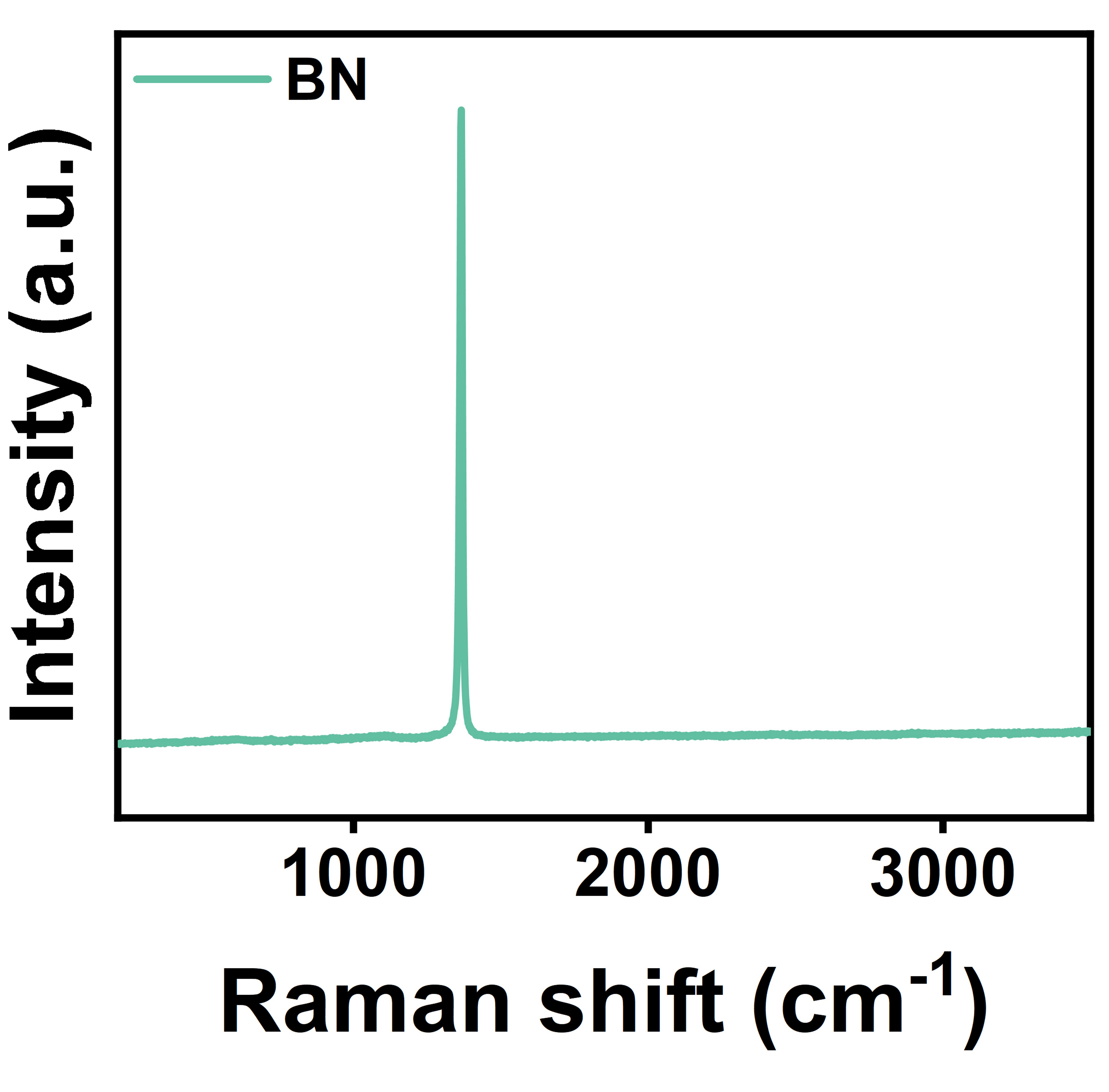


**Figure S10.** The Raman spectra of BN.


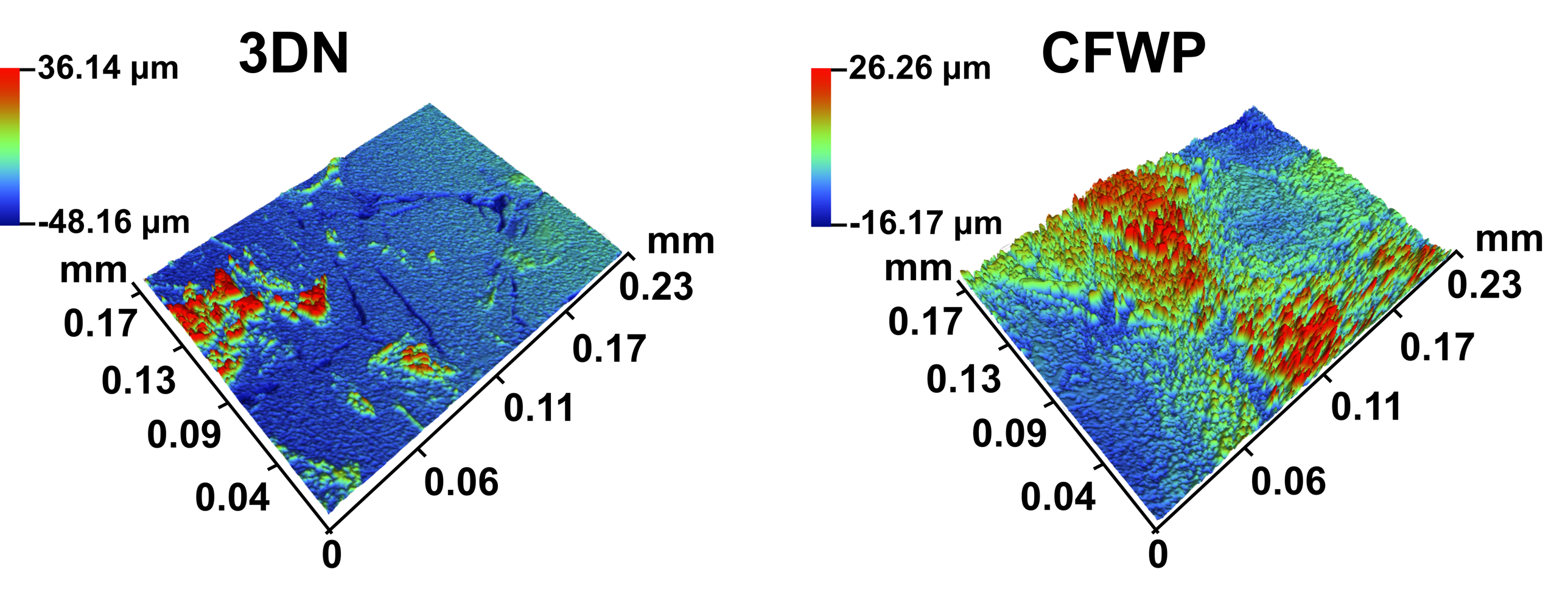


**Figure S11.** 3D optical profile images of 3DN and CFWP.


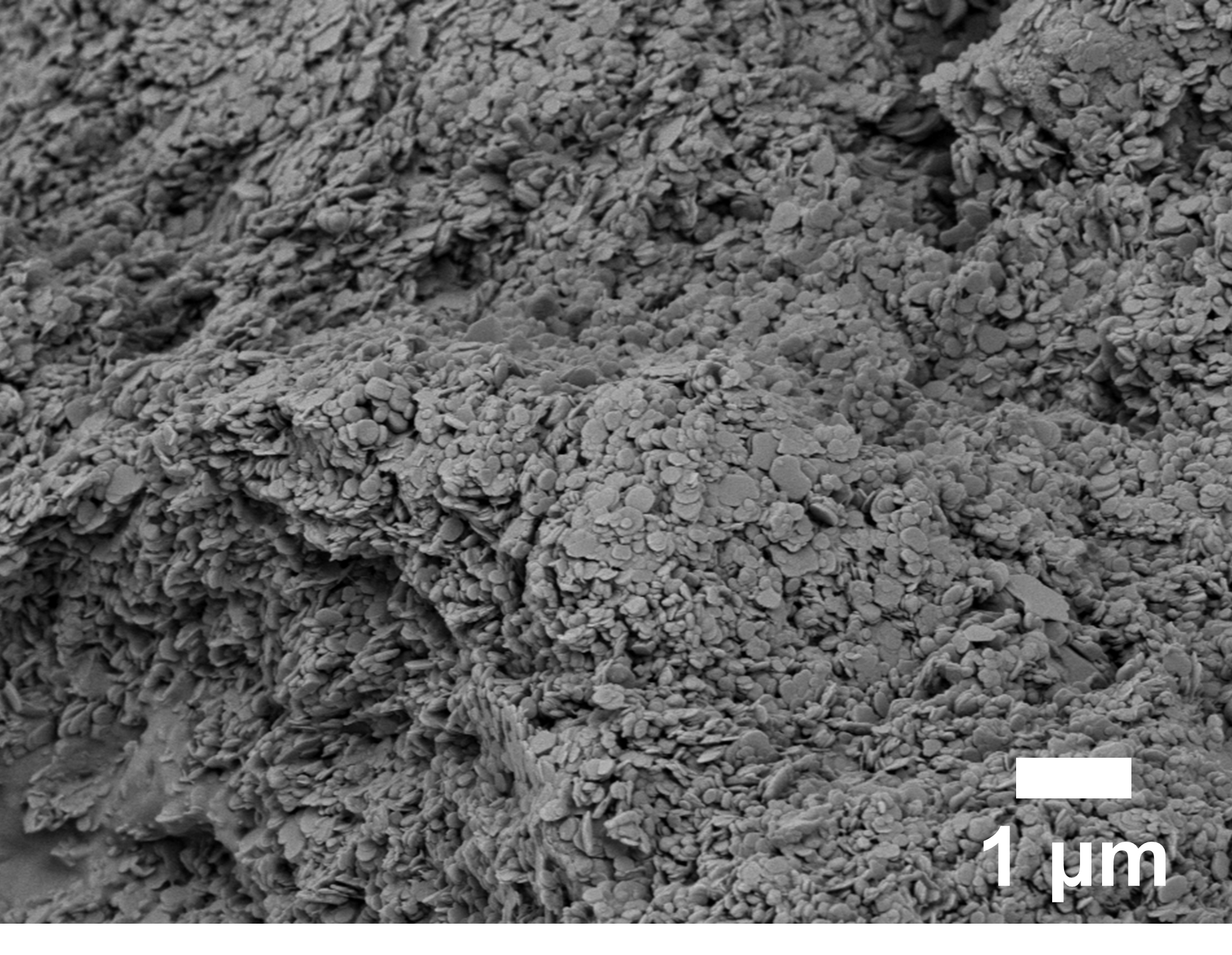


**Figure S12.** The cross-sectional SEM image of NCF-30.





**Figure S13.** (a) Stress-strain curves of 3DN and 3DN@BNNS. (b) Hhistogram of strain, stress, and Young’s modulus of CFWP, NCF-0, NCF-10, NCF-20, and NCF-30. *P < 0.05, **P < 0.01, ***P < 0.001, ****P < 0.0001.


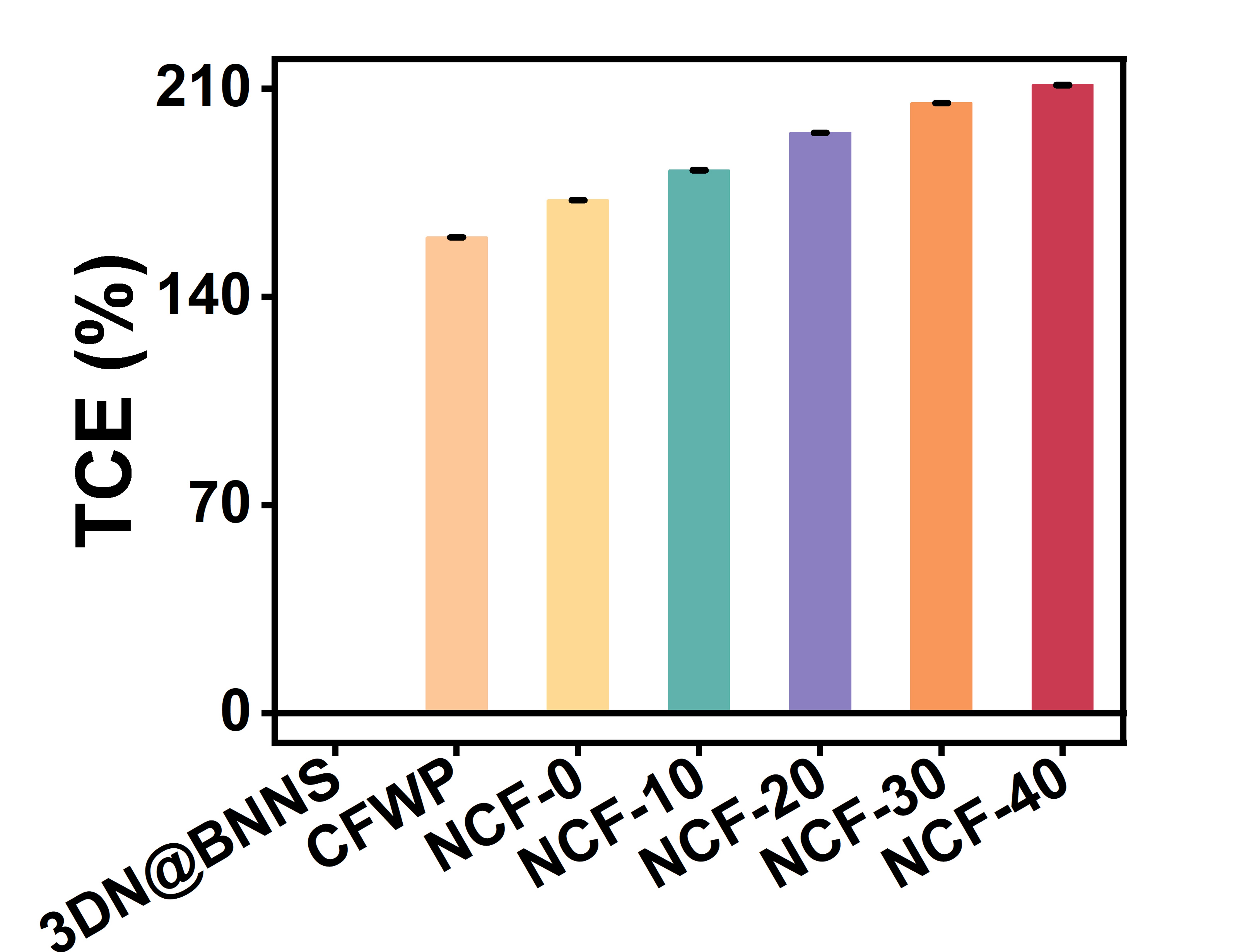


**Figure S14.** TCE values of CFWP, NCF-0, NCF-10, NCF-20, NCF-30, and NCF-40 relative to that of 3DN@BNNS.





**Figure S15.** (a) TG and (b) DTG curves of NCF-0, NCF-10, NCF-20, NCF-30, and NCF-40.


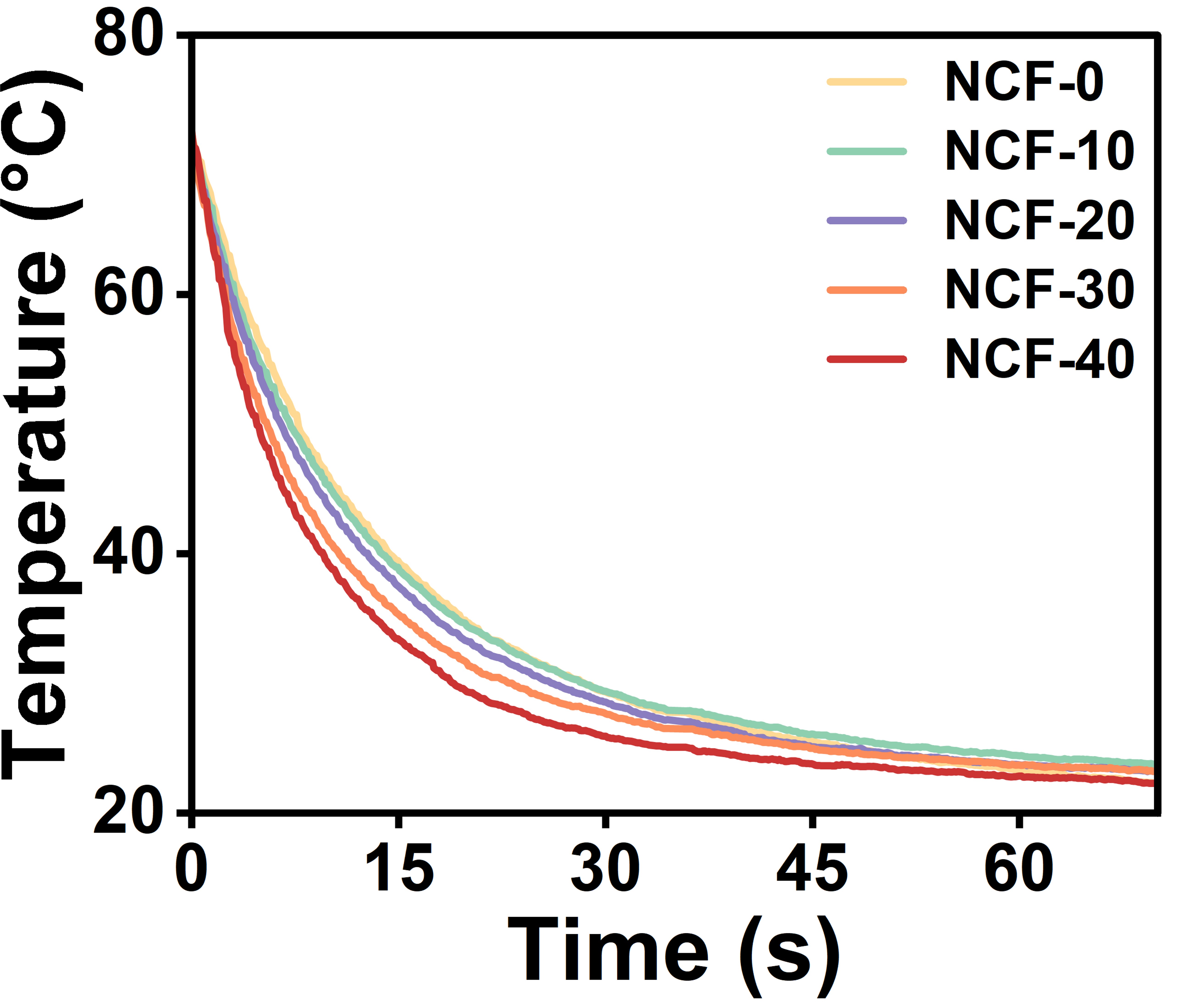


**Figure S16.** The cooling performance of NCF-0, NCF-10, NCF-20, NCF-30, and NCF-40.

.


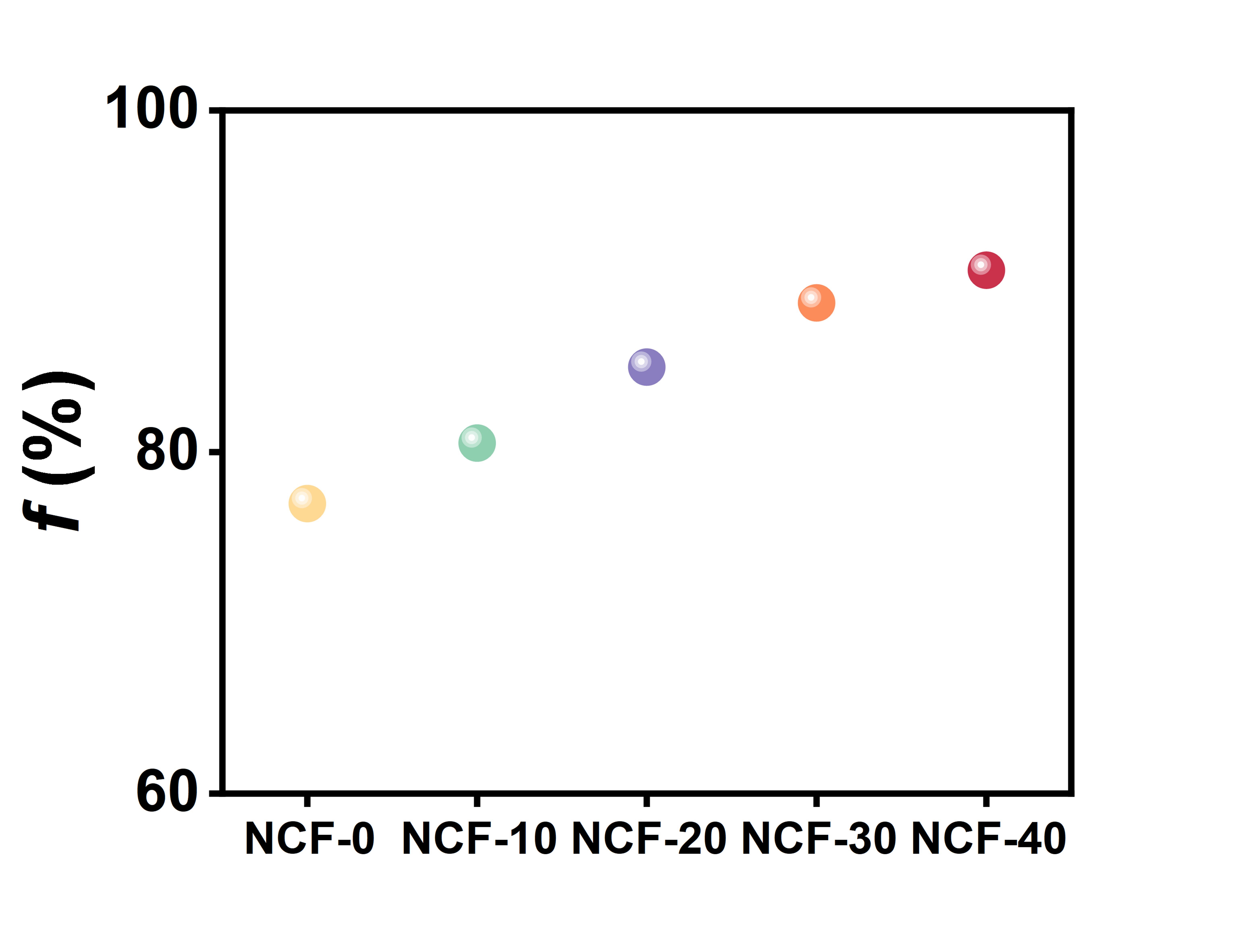


**Figure S17.** The synergistic effect index (*f*) of NCF-0, NCF-10, NCF-20, NCF-30, and NCF-40.


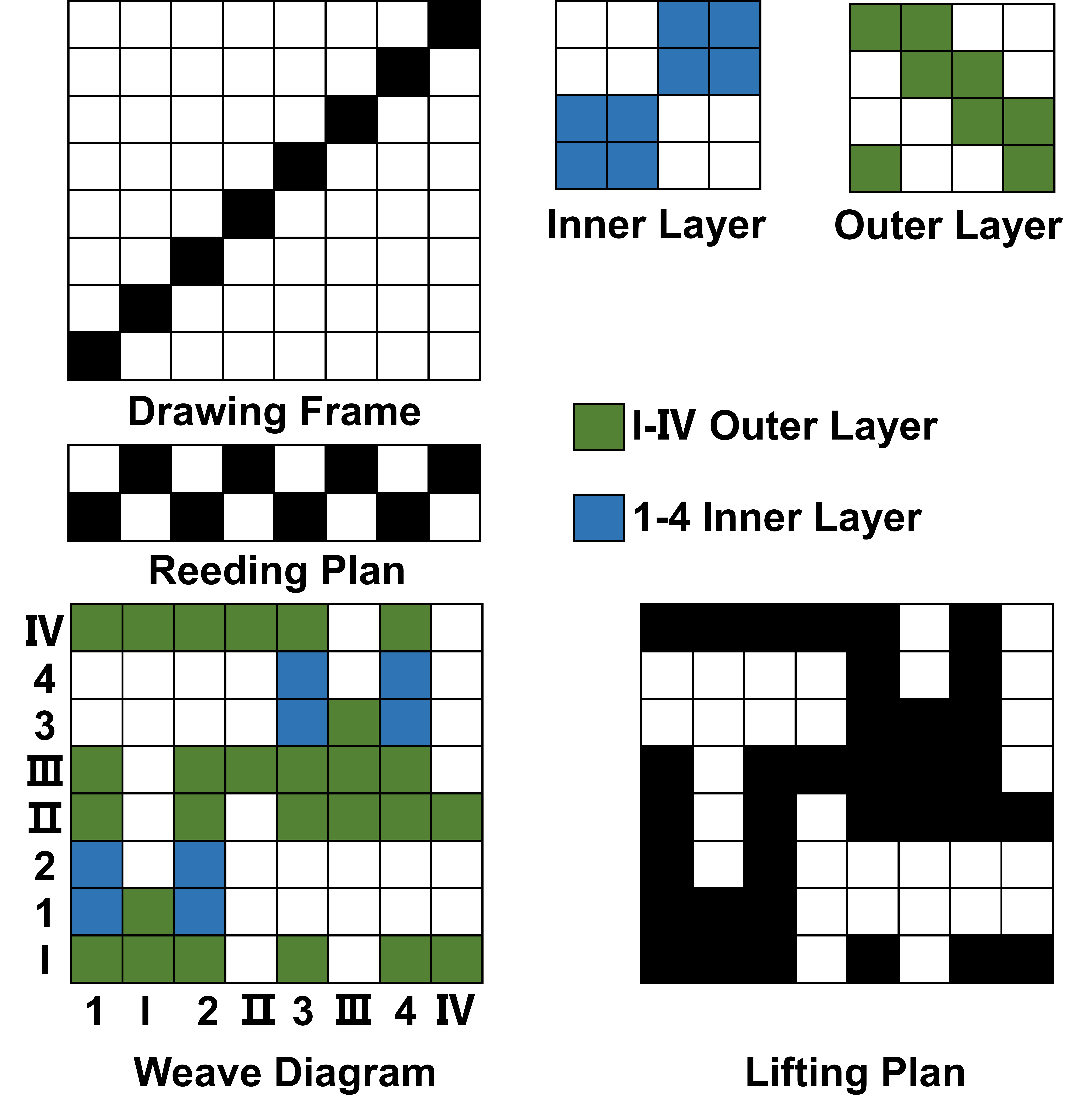


**Figure S18.** Schematic of the double-layer woven textile structure.


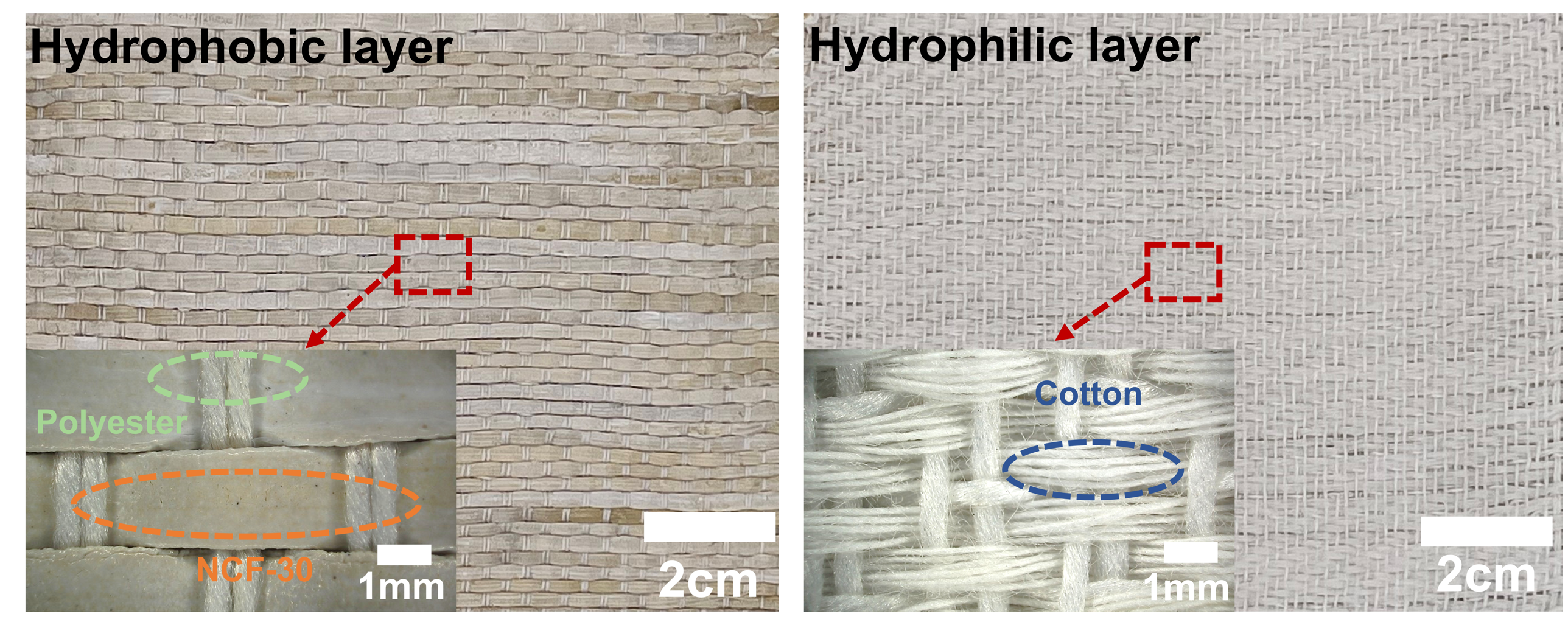


**Figure S19.** Optical images of the hydrophobic layer and hydrophilic layer of WCT.


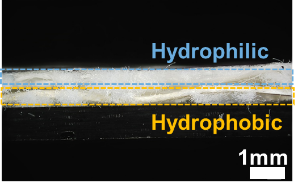


**Figure S20.** Side view of WCT.


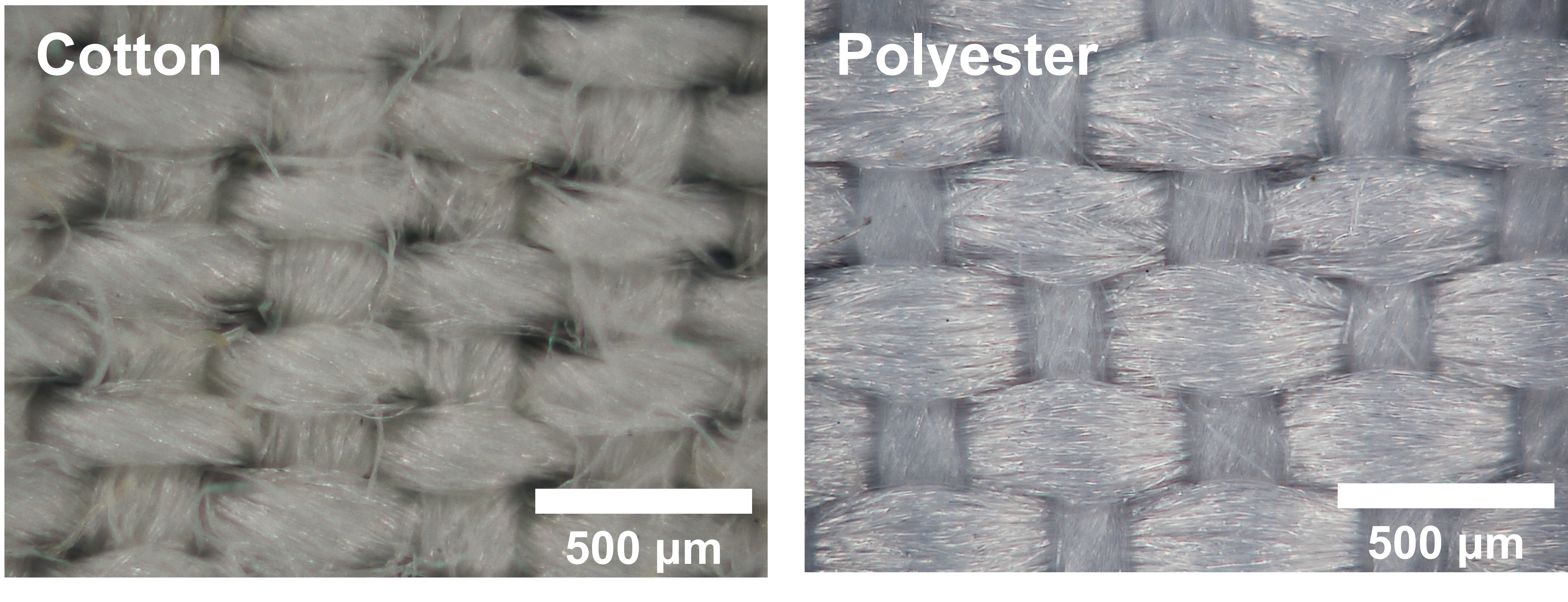


**Figure S21.** Optical images of the commerial cotton and polyester fabrics.


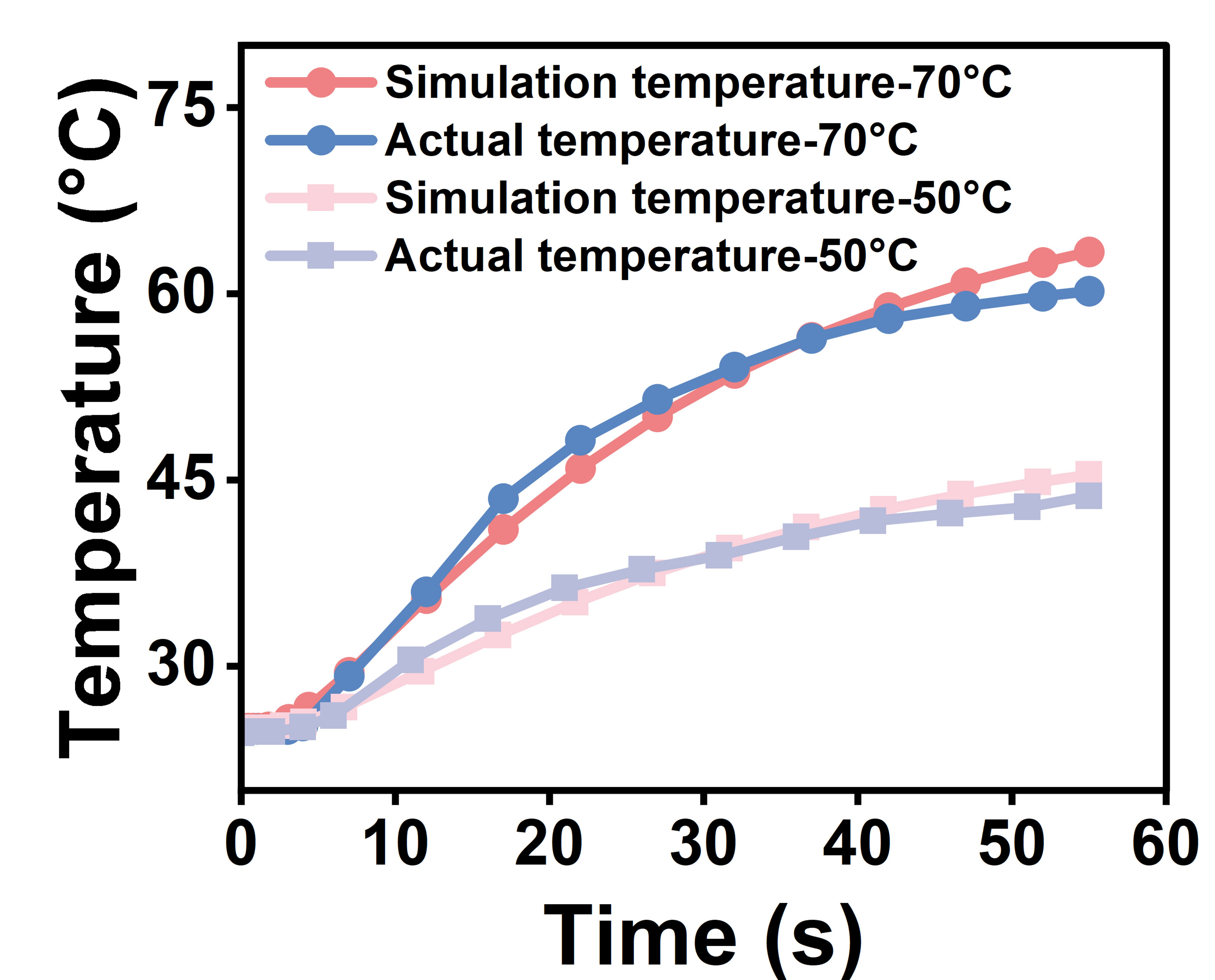


**Figure S22.** Comparsion of temperature response between simulation and actual environment.


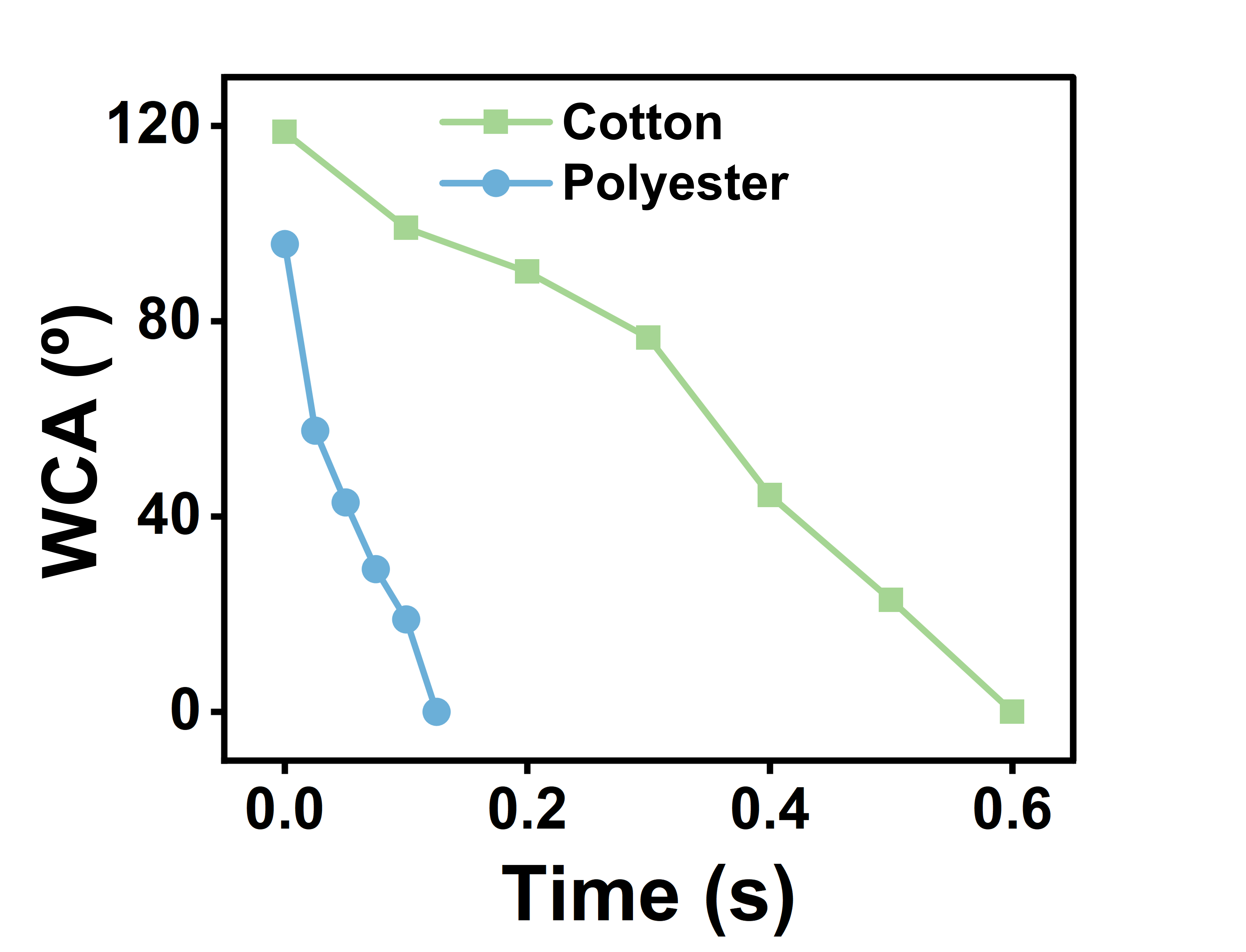


**Figure S23.** WCA of cotton and polyester yarns used in WCT.





**Figure S24.** Moisture management tester for the directional water transport capacity test.


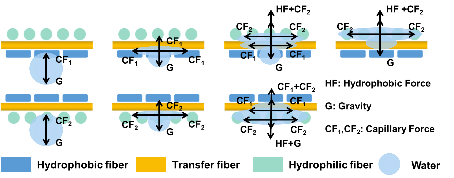


**Figure S25.** Illustration of the directional perspiration mechanism.


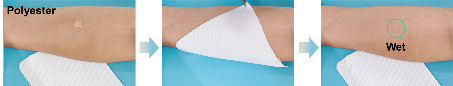


**Figure S26.** Photographs of polyester textile before and after simulated sweating.
